# Ca^2+^ dysregulation in cardiac stromal cells sustains fibro-adipose remodeling in Arrhythmogenic Cardiomyopathy and can be modulated by flecainide

**DOI:** 10.1186/s12967-022-03742-8

**Published:** 2022-11-12

**Authors:** Angela S. Maione, Pawan Faris, Lara Iengo, Valentina Catto, Luca Bisonni, Francesco Lodola, Sharon Negri, Michela Casella, Anna Guarino, Gianluca Polvani, Marina Cerrone, Claudio Tondo, Giulio Pompilio, Elena Sommariva, Francesco Moccia

**Affiliations:** 1 Unit of Vascular Biology and Regenerative Medicine, Centro Cardiologico Monzino IRCCS, Via Parea 4, 20138 Milan, Italy; 2 Department of Biology and Biotechnology “Lazzaro Spallanzani”, University of Pavia, Pavia, Italy; 3 Department of Clinical Electrophysiology and Cardiac Pacing, Centro Cardiologico Monzino IRCCS, Milan, Italy; 4 Laboratory of Cardiac Cellular Physiology, Department of Biotechnology and Bioscience, University of Milano-Bicocca, Milan, Italy; 5 Cardiology and Arrhythmology Clinic, University Hospital “Umberto I-Salesi-Lancisi”, Ancona, Italy; 6 Cardiovascular Tissue Bank of Lombardy, Centro Cardiologico Monzino IRCCS, Milan, Italy; 7 Medicine, Leon H. Charney Division of Cardiology, Heart Rhythm Center and Cardiovascular Genetics Program, New York University School of Medicine, New York, USA; 8 Department of Biomedical, Surgical and Dentist Sciences, University of Milano, Milan, Italy

**Keywords:** Arrhythmogenic cardiomyopathy, Cardiac mesenchymal stromal cells, Calcium signalling, CaMKII, Store-operated Ca^2+^ entry, Flecainide

## Abstract

**Background:**

Cardiac mesenchymal stromal cells (C-MSC) were recently shown to differentiate into adipocytes and myofibroblasts to promote the aberrant remodeling of cardiac tissue that characterizes arrhythmogenic cardiomyopathy (ACM). A calcium (Ca^2+^) signaling dysfunction, mainly demonstrated in mouse models, is recognized as a mechanism impacting arrhythmic risk in ACM cardiomyocytes. Whether similar mechanisms influence ACM C-MSC fate is still unknown.

Thus, we aim to ascertain whether intracellular Ca^2+^ oscillations and the Ca^2+^ toolkit are altered in human C-MSC obtained from ACM patients, and to assess their link with C-MSC-specific ACM phenotypes.

**Methods and results:**

ACM C-MSC show enhanced spontaneous Ca^2+^ oscillations and concomitant increased Ca^2+^/Calmodulin dependent kinase II (CaMKII) activation compared to control cells. This is manly linked to a constitutive activation of Store-Operated Ca^2+^ Entry (SOCE), which leads to enhanced Ca^2+^ release from the endoplasmic reticulum through inositol-1,4,5-trisphosphate receptors. By targeting the Ca^2+^ handling machinery or CaMKII activity, we demonstrated a causative link between Ca^2+^ oscillations and fibro-adipogenic differentiation of ACM C-MSC. Genetic silencing of the desmosomal gene *PKP2* mimics the remodelling of the Ca^2+^ signalling machinery occurring in ACM C-MSC. The anti-arrhythmic drug flecainide inhibits intracellular Ca^2+^ oscillations and fibro-adipogenic differentiation by selectively targeting SOCE.

**Conclusions:**

Altogether, our results extend the knowledge of Ca^2+^ dysregulation in ACM to the stromal compartment, as an etiologic mechanism of C-MSC-related ACM phenotypes. A new mode of action of flecainide on a novel mechanistic target is unveiled against the fibro-adipose accumulation in ACM.

**Supplementary Information:**

The online version contains supplementary material available at 10.1186/s12967-022-03742-8.

## Introduction

Arrhythmogenic cardiomyopathy (ACM, also indicated as arrhythmogenic right ventricular cardiomyopathy, ARVC) is a rare cardiac disease of genetic origin characterized by a high incidence of sudden death in young people, due to malignant arrhythmic events, and by progressive degeneration of the ventricular myocardium, mainly the right ventricle, leading to heart failure. Mutations causing ACM are mostly found in genes encoding for desmosomal proteins, such as Plakophilin2 (*PKP2)*, Plakoglobin (*JUP)*, Desmoplakin (*DSP)*, Desmoglein-2 (*DSG2)*, Desmocollin-2 (*DSC2)* [1]. Mutated desmosomes are responsible for alterations in mechanical stability, electrical coupling between cells, and cellular signalling that likely lead to cardiomyocyte (CM) death and to the increase of adipogenic and fibrogenic genes expression for aberrant repair [2, 3]. The progressive loss of ventricular myocardium and the fibro-fatty infiltration contribute to determine clinical events such as ventricular arrhythmias and impaired ventricular systolic function. A desmosome-dyad axis dysfunction, mainly demonstrated in mouse models, is recognized as a mechanism impacting arrhythmic risk in ACM CM [4–10].

Different studies revealed the important contribution of non-myocyte cells, such as Cardiac Mesenchymal Stromal Cells (C-MSC), to the ACM pathogenic mechanisms. C-MSC support the structural and functional integrity of the myocardium. They can be affected by ACM causative mutations based on the expression of desmosomal genes and they are the source of adipocytes and myofibroblasts in ACM patients’ hearts [11, 12].

MSC differentiation process can be influenced by spontaneous oscillations in intracellular calcium concentration ([Ca^2+^]_i_) that are frequently detected in these cell types [13, 14]. The frequency of Ca^2+^ oscillations is different between undifferentiated MSC and MSC undergoing differentiation. Furthermore, the Ca^2+^ spiking pattern can vary depending on the differentiation level and type [13, 15, 16].

The mechanisms driving spontaneous Ca^2+^ oscillations were mainly investigated in adipose tissue- and bone marrow-derived MSC, in which they are sustained by both Ca^2+^ release from endoplasmic reticulum (ER) and Ca^2+^ inflow from extracellular space [17]. Endogenous Ca^2+^ is liberated from the ER through inositol-1, 4, 5-trisphosphate (IP3) receptors (IP3R), whereas ryanodine receptors (RYR) are not expressed in most of MSC. Depletion of the Ca^2+^ ER store results in the activation of “store-operated” Ca^2+^ channels (SOCC) on the plasma membrane (PM), which refills the ER with Ca^2+^ in a Sarco-Endoplasmic Reticulum Ca^2+^-ATPase (SERCA)-dependent manner [17]. SOCC depend on the physical interaction between STIM1-2, which serve as the ER Ca^2+^ sensor engaged upon ER Ca^2+^ depletion, and ORAI1-3, which form the Ca^2+^-permeable channel on the PM. In human MSC, SOCC impinge on STIM1 and ORAI1 and are required to maintain the spontaneous oscillations in [Ca^2+^]_i_ that regulate differentiation [18]. Additional pathways for extracellular Ca^2+^ entry are through L-type voltage-operated Ca^2+^ channels (VOCC) [19, 20]. Intriguingly, Store-operated Ca^2+^ entry (SOCE) is constitutively activated in several genetic muscle disorders, thereby enhancing resting [Ca^2+^]_i_ and increasing the ER Ca^2+^ load [21, 22]. The mechanisms that regulate spontaneous Ca^2+^ oscillations in C-MSC, their outcome on fibro-adipogenic differentiation and, therefore, their potential involvement in ACM, are currently unknown.

A molecular decoder of Ca^2+^ oscillations is the Ca^2+^/Calmodulin-dependent protein kinase II (CaMKII), a ubiquitous enzyme that mediates the Ca^2+^ effects on cellular targets involved in multiple cellular functions. CaMKII can act as a frequency decoder of Ca^2+^ oscillations in vitro.

This property is critically dependent on the effectiveness of individual Ca^2+^ spikes in stimulating autophosphorylation of the enzyme. These oscillations can be decoded according to their frequency, amplitude, duration and number [23].

The regulation of Ca^2+^-handling proteins by CaMKII [24, 25] leads to an increase in spontaneous Ca^2+^-release from the Sarcoplasmic Reticulum (SR) [26]. As consequence, in pathological scenarios, these events result in increased Ca^2+^ in the cytosol. Basically, CaMKII is a pro-arrhythmogenic protein in the heart, but its role in non-myocyte cells, e.g., C-MSC, is still unknown. Intriguingly, CaMKII also regulates fate decisions in multiple stem cell types [27, 28], including MSC [29], but the mechanistic link between intracellular Ca^2+^ oscillations, CaMKII activation, and stem cell differentiation is still unclear.

Based on these premises, we have hypothesized a correlation between intracellular Ca^2+^ oscillations and the fibro-adipogenic process in the ACM pathogenesis. We exploited a multidisciplinary approach to perform in vitro experiments on primary human C-MSC obtained from ACM patients.

Our findings show for the first time that the increased frequency of spontaneous Ca^2+^ oscillations, as well as the resulting CaMKII activation, are responsible for the ACM aberrant lipid/fibrotic accumulation and provide the first proof-of-concept that this signalling pathway can be targeted for therapeutic purposes.

## Materials and methods

### Ethical statement

This study complies with the declaration of Helsinki and was approved by the Centro Cardiologico Monzino Ethics Committee (R1020/19-CCM1072; date of approval: 3/7/2019). Written consent was signed by participating ACM patients (right ventricle endomyocardial biopsy samples and blood samples). The healthy control (HC) right ventricle endomyocardial samples were obtained from cadaveric donors from the “Cardiovascular Tissue Bank” of Centro Cardiologico Monzino (MTA signed 5 November 2019). The Additional file 1: Tables S1-S2 summarize the clinical and genetic features of the enrolled ACM and HC subjects, respectively. Different C-MSC from ACM and HC were used for the in vitro experiments depending on availability and culture passage number.

### C-MSC isolation and culture

C-MSC were isolated and cultured as previously reported [11, 30]. Briefly, ventricular samples were washed with PBS, cut into 2–3 mm pieces, and incubated at 37 °C for 1.5 h under continuous agitation in Iscove’s modified Dulbecco’s media (IMDM; Gibco, Waltham, Massachusetts, USA) containing 3 mg/ml collagenase NB4 (Serva, Heidelberg, Germany). The digested solution was then centrifuged at 400 g for 10 min, washed with PBS, and centrifuged again. The obtained pellet was resuspended in growing medium (GM), consisting of IMDM supplemented with 20% fetal bovine serum (FBS; Euroclone, Milan, Italy), 10 ng/ml basic fibroblast growth factor (R&D Systems, Minneapolis, Canada), 10,000 U/ml penicillin (Invitrogen, Carlsbad, California, USA), 10,000 µg/ml streptomycin (Invitrogen, Carlsbad, California, USA), and 20 mmol/l L-Glutamine (Sigma-Aldrich, St. Louis, Missouri, USA). The cells were seeded onto uncoated Petri dishes (Corning, Corning, New York, USA). Non-adherent cells were removed after 24 h.

### Differentiation of C-MSC

To prompt the adipogenic differentiation, C-MSC were plated at concentration of 30.000 cells/cm^2^ and cultured in adipogenic medium (AM), consisting of IMDM supplemented with 10% FBS (Euroclone, Milan, Italy), 0.5 mmol/l 3-isobutyl-1-methylxanthine (SigmaAldrich, St. Louis, Missouri, USA), 1 µmol/l hydrocortisone (SigmaAldrich, St. Louis, Missouri, USA), 0.1 mmol/l indomethacin (Sigma-Aldrich, St. Louis, Missouri, USA), 10,000 U/ml penicillin (Invitrogen, Carlsbad, California, USA), 10,000 µg/ml streptomycin (Invitrogen, Carlsbad, California, USA), and 20 mmol/l L-Glutamine (Sigma-Aldrich, St. Louis, Missouri, USA) as previously described [30].

To stimulate pro-fibrotic differentiation, C-MSC were plated at concentration of 30.000 cells/cm^2^ cells and treated with 5 ng/mL of TGF-β1 (PeproTech, London, UK), after overnight (O/N) growth in low serum GM (2% FBS) as previously described [12].

### Flow cytometry

To determine the lipid accumulation, cells were detached with TrypLE Select (Life Technologies, Carlsbad, California, USA) and stained using 12.5 ng/ml of Nile Red (Invitrogen, Carlsbad, California, USA), to mark intracellular neutral lipids. After washing with PBS to ensure the removal of unbound dye, quantitative results were obtained by evaluating Nile Red fluorescence with FACS Gallios (Beckman Coulter, Brea, California, USA).

### PKP2 silencing

HC C-MSC were plated at a density of 12.500 cell/cm^2^ in GM and transduced with pooled lentiviral particles containing shRNAs targeting both variants of human *PKP2* (Gene ID 5381) in psi-LVRU6GP (with U6 promoter, eGFP reporter, puromycin resistance; Genecopoeia; Rockville, Maryland) or with the correspondent scrambled control lentiviral particles (Genecopoeia; Rockville, Maryland) for 24 h. After checking the transduction efficiency by detection of the GFP signal, 2 lg/ml puromycin was added to select transduced cells. After cell amplification, PKP2 reduction was assayed by Western blot (WB).

### mRNA extraction and qRT-PCR assay

Cell cultures were lysed in RL lysis buffer (Norgen Biotek corp., Thorold, Canada). RNA was isolated from cells by using a Total RNA Purification kit (Norgen Biotek corp., Thorold, Canada). The quantification of the isolated RNA was determined by NanoDrop spectrophotometer (ND-1000, EuroClone, Milan, Italy). Reverse transcription was conducted with SuperScript III (Invitrogen, Carlsbad, CA, USA) following the manufacturer’s instructions. qRT-PCR was performed with the use of the iQTM SYBR Green Super Mix (Bio-Rad Laboratories, Hercules, CA, USA) and specific primers (reported in Additional file 1: Table S3). All reactions were performed in a 96-well format with the 7900HT Fast Real-Time PCR System (Thermo Fisher Scientific, Massachusetts, USA). The relative quantities of specific mRNA were obtained with the use of the comparative Ct method and were normalized to the housekeeping gene glyceraldehyde 3-phosphate dehydrogenase (GAPDH).

### Protein extraction and western blot analysis

C-MSC were lysed in cell lysis buffer (Cell Signaling Technology, Danvers, MA, USA) supplemented with protease and phosphatase inhibitor cocktails (Sigma-Aldrich, Saint Louis, MO, USA). Total protein extracts were subjected to SDS-PAGE and transferred onto a nitrocellulose membrane (Bio-Rad, California, USA). The membranes were blocked for 1 h at room temperature in 5% non-fat dry milk in Wash Buffer (Tris Buffer Sulfate, 0.1% Tween-20) and then incubated O/N at 4 °C with the appropriate primary antibodies (reported in Additional file 1: Table S4). The membranes were incubated with peroxidase-conjugated secondary antibodies (GE Healthcare, Chicago, IL, USA) for 1 h. Signals were visualized using the LiteUP Western Blot Chemiluminescent Substrate (EuroClone, Milan, Italy). Images were acquired with the ChemiDoc™ MP Imaging System (Bio-Rad, California, USA), and densitometric analysis of membranes was performed using the ImageJ software (National Institutes of Health, Bethesda, MD, USA). C-MSC proteins were normalized according to Histone H3 or alternatively GAPDH and TUBULIN based on the gel gradient used.

### Immunofluorescence analysis

For cell immunofluorescence, cells were fixed using 4% paraformaldehyde (Santa Cruz biotechnology, Texas, US) for 10 min. After blocking unspecific binding sites, with PBS supplemented with 5% BSA and 0.1% Triton X-100 (PBS-T/BSA) for 60 min, the slides were incubated with specific primary antibody for Collagen (as reported in Additional file 1: Table S4) O/N at 4 °C. Fluorescence-labeled secondary antibodies (Invitrogen, Carlsbad, CA, USA) were added for 1 h at room temperature (RT). Otherwise, fixed cells were stained using 12.5 ng/ml of Nile Red (Invitrogen, Carlsbad, California, USA) for 1 h at RT. Nuclei were stained with Hoechst 33,342 (Sigma-Aldrich, Saint Louis, MO, USA). Images were acquired with a confocal microscope in Z-stack mode with 40 × oil immersion objective (Zeiss LSM710-ConfoCor3 LSM, Zeiss, Germany) using the software Zen 2008 (Zeiss, Germany). Fluorescence signal quantification was performed using ImageJ software on Z-Stacks images. Single channels from each image were converted into 8-bit grayscale images and thresholded in order to subtract background. The fluorescence value has been normalized to the number of nuclei per field (at least 5 for each experiment). Nuclei counting was performed using the ImageJ tool.

### Solutions for intracellular Ca^2+^ recordings

Physiological salt solution (PSS) had the following composition (in mM): 150 NaCl, 6 KCl, 1.5 CaCl2, 1 MgCl2, 10 Glucose, 10 Hepes. In Ca^2+^-free solution (0Ca^2+^), Ca^2+^ was substituted with 2 mM NaCl, and 0.5 mM EGTA was added. Solutions were titrated to pH 7.4 with NaOH. In Mn^2+^-quenching experiments, 200 mM MnCl_2_ was added to the 0Ca^2+^ external solution. For high- K^+^, extracellular solution was prepared by replacing 100 mM NaCl with an equimolar amount of KCl. The osmolality of PSS as measured with an osmometer (Wescor 5500, Logan, UT) was 338 mmol/kg.

### [Ca^2+^]_i_ measurements

C-MSC were loaded with 2 μM Fura-2 acetoxymethyl ester (Fura-2/AM; 1 mM stock in dimethyl sulfoxide) in PSS for 30 min at 37 °C. After washing in PSS, the coverslip was fixed to the bottom of a Petri dish and the cells were observed by an upright epifluorescence Axiolab microscope (Carl Zeiss, Oberkochen, Germany), usually equipped with a Zeiss × 40 Achroplan objective (water immersion, 2.0 mm working distance, 0.9 numerical aperture). C-MSC were excited alternately at 340 and 380 nm, and the emitted light was detected at 510 nm. A first neutral density filter (1 or 0.3 optical density) reduced the overall intensity of the excitation light and a second neutral density filter (optical density = 0.3) was coupled to the 380 nm filter to approach the intensity of the 340 nm light. A round diaphragm was used to increase the contrast. The excitation filters were mounted on a filter wheel (Lambda 10, Sutter Instrument, Novato, CA, USA). Custom software, working in the LINUX environment, was used to drive the camera (Extended-ISIS Camera, Photonic Science, Millham, UK) and the filter wheel, and to measure and plot online the fluorescence from 10 up to 100 rectangular “regions of interest” (ROI). Each ROI was identified by a number. Since cell borders were not clearly identifiable, a ROI may not include the whole cell or may include part of an adjacent cell. Adjacent ROIs never superimposed. [Ca^2+^]_i_ was monitored by measuring, for each ROI, the ratio of the mean fluorescence emitted at 510 nm when exciting alternatively at 340 and 380 nm (shortly termed “ratio”). An increase in [Ca^2+^]_i_ causes an increase in the ratio. Ratio measurements were performed and plotted on-line every 3 s. Resting [Ca^2+^]_i_ in HC and ACM C-MSC was evaluated by exploiting the Grynkiewicz methods. The experiments were performed at RT (22 °C).

Resting Ca^2+^ entry in HC and ACM C-MSC was investigated by using the Mn^2+^-quenching technique. Mn^2+^ may quench Fura-2/AM fluorescence and cannot be extruded from the cytoplasm by the Ca^2+^ transporting system located either on the plasma membrane or in intracellular organelles. Since Mn^2+^ permeates the cells via SOCC, the rate of Fura-2/AM quenching by Mn^2+^ is regarded as an index of basal SOCE activation [31]. Experiments were carried out at the 360 nm wavelength, the isosbestic wavelength for Fura-2/AM, and in a 0Ca^2+^ extracellular solution to avoid Ca^2+^ competition for Mn^2+^ entry and enhance Mn^2+^ quenching, as described in [30].

### Statistical analyses

Quantitative results are expressed as mean ± SEM. Statistical analysis was performed with GraphPad Prism 9. Quantitative variables were analyzed by one-way ANOVA with Dunnett’s post-test or Two-tailed Student’s t-test, as appropriate. Categorical variables were compared with Fisher’s exact test. A value of p ≤ 0.05 was considered statistically significant.

Spontaneous Ca^2+^ oscillations in HC and ACM C-MSC were recorded for 1 h and the following parameters evaluated: amplitude of the 1^st^ Ca^2+^ spike and frequency of the spiking signal (i.e., number of transients/1 h), as shown in [32, 33]. The amplitude of Ca^2+^ release in response to either CPA or ATP was measured as the difference between the ratio at the peak of intracellular Ca^2+^ mobilization and the mean ratio of 1 min baseline before the peak. The magnitude of SOCE evoked by either CPA or ATP upon Ca^2+^ restoration to the bath was measured as the difference between the ratio at the peak of extracellular Ca^2+^ entry and the mean ration of 1 in baseline before Ca^2+^ re-addition. The rate of Mn^2+^ influx was evaluated by measuring the slope of the fluorescence intensity curve at 400 s after Mn^2+^ addition. Each experimental series was performed on C-MSC deriving from at least three different HC donors and three different ACM patients.

## Results

### Intracellular Ca^2+^ oscillations and CaMKII phosphorylation are enhanced in ACM C-MSC

**Fig. 1:**
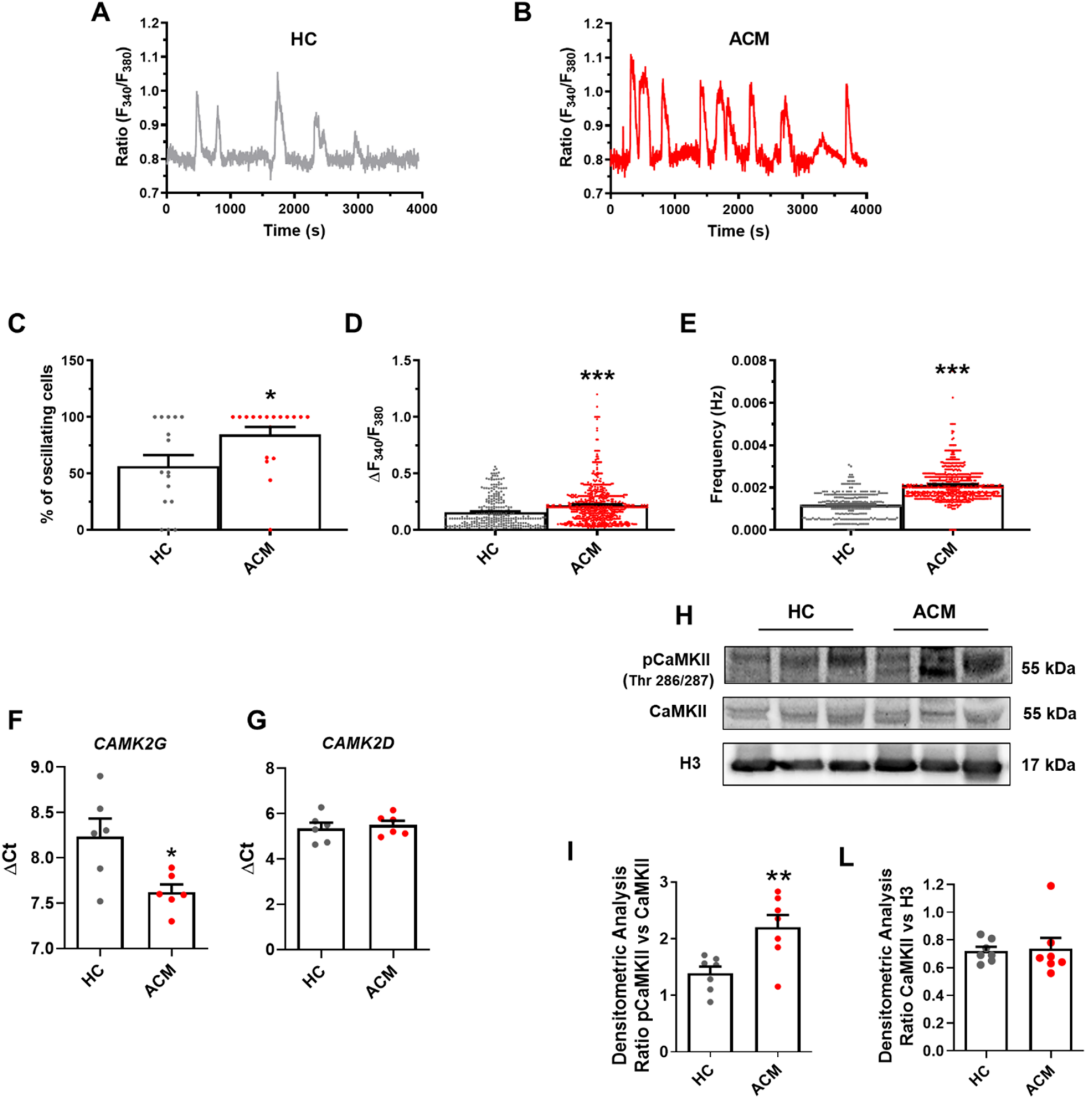
Spontaneous Ca^2+^ oscillations and CaMKII activation are enhanced in human-derived ACM C-MSC. **A**–**B** Representative Ca^2+^ traces in C-MSC from HC donors and ACM patients loaded with Fura-2/AM. The measurements were performed on unstimulated cells cultured in GM. **C**–**E** The graphs represent: **C** percentage C-MSC displaying spontaneous Ca^2+^ oscillations (n = 567 cells; Two-tailed Student’s t-tests); **D** oscillation amplitude (n = 294 cells HC *vs.* n = 634 cells ACM; Two-tailed Student’s t-tests) and **E** oscillation frequency (n = 294 cells HC *vs.* n = 639 cells ACM; Two-tailed Student’s t-tests). **F**–**G** Expression of *CAMK2G* and *CAMK2D* isoforms in total RNA extracts of C-MSC from HC donors and ACM patients. GAPDH was used as a house-keeping gene and qRT-PCR data are presented as the genes threshold cycles (Ct) with respect to the housekeeping gene GAPDH (ΔCt) (n = 6 biological replicates; Two-tailed Student’s t-tests). **H** Representative images of WB analysis of proteins extracted from ACM and HC C-MSC cultured in GM, hybridized with anti-pCaMKII and anti-CaMKII antibodies. Immunostaining of the housekeeping H3 is shown for normalization. **I**–**L** Densitometric analysis of pCaMKII (n = 8 biological replicates) and CaMKII (n = 8 biological replicates) levels, normalized on H3 (Two-tailed Student’s t-tests). Data information: mean ± SEM. *P < 0.05, **P < 0.01 and ***P < 0.0001

We characterized spontaneous Ca^2+^ oscillations in HC and ACM C-MSC loaded with the Ca^2+^-sensitive fluorophore, Fura-2/AM. Resting [Ca^2+^]_i_ was significantly higher in ACM C-MSC compared to the HC cells (Additional file 1: Figure S1). Furthermore, spontaneous Ca^2+^ oscillations **(**Fig. 1A, B) arise in a significantly higher percentage (*n* = 567 cells; HC 56.38% ± 9.746 *vs. n* = 729 cells; ACM 84.62% ± 6.553; *P* = 0.0198; Fig. 1C) and display a significantly higher amplitude (*n* = 294; HC 0.1574 ± 0.008000 a.u. *vs. n* = 634; ACM 0.2202 ± 0.006652 a.u.; *P* < 0.0001; Fig. 1D) and frequency (*n* = 294; HC 0.001184 ± 3.372e-005 Hz *vs. n* = 634; ACM 0.002128 ± 3.006e-005 Hz; *P* < 0.0001; Fig. 1E) in ACM C-MSC. In MSC deriving from other tissues, such as the bone marrow, spontaneous Ca^2+^ oscillations are driven by autocrine/paracrine release of ATP, which signals through the G_q_-Protein Coupled P2Y_1_ receptors [34, 35]. In agreement with these observations, both suramin, a non-selective P2Y receptor antagonist [36], and MRS-2179, a specific P2Y_1_ blocker [37] inhibited the spontaneous Ca^2+^ oscillations in the majority of ACM C-MSC, whereas they significantly reduced both the amplitude and frequency of the spiking Ca^2+^ signal in the remaining cells (Additional file 1: Figure S2).

Since CaMKII is a key cellular effector of intracellular Ca^2+^ oscillations [23, 38], we evaluated the presence and activation of CaMKII in C-MSC. CaMKII consists of four separate isoforms with different tissue distribution [39]. qRT-PCR analysis confirmed that *CAMK2G* (*n* = 6; HC 8.235 ± 0.1982 *vs.* ACM 7.620 ± 0.08473; *P* = 0.0171; Fig. 1F) and *CAMK2D* (*n* = 6; HC 5.350 ± 0.2509 *vs.* ACM 5.493 ± 0.1893; *P* = 0.6581; Fig. 1G) transcripts, predominant isoforms in the heart [40, 41], were highly detectable in C-MSC whereas *CAMK2A* and *CAMK2B* were barely expressed (Additional file 1: Figure S3). We further assessed the activation of CaMKII by using a specific antibody that recognizes the phosphorylated (active) form of the protein. WB analysis revealed that CaMKII was highly phosphorylated in ACM C-MSC compared to HC C-MSC (*n* = 8; HC 0.9286 ± 0.06519 *vs.* ACM 1.840 ± 0.3872; *P* = 0.0387; Fig. 1H, I). The densitometric analysis of total form of CaMKII confirmed that the protein was expressed in our cellular system and its abundance was similar in ACM and HC cells (*n* = 8; HC 0.7200 ± 0.03071 *vs.* ACM 0.7357 ± 0.07973; *P* = 0.8571; Fig. 1H, L). A detailed characterization of the underlying signalling mechanisms revealed the enhanced Ca^2+^ oscillations driving CaMKII hyperactivation in ACM C-MSC are driven by SOCE and IP3R (detailed in Additional file 1: text and Figure S4-S8).

### SOCE is constitutively activated in ACM C-MSC

The enhancement in spontaneous Ca^2+^ activity could reflect an increase in IP3-induced ER Ca^2+^ release and/or SOCE activity in ACM C-MSC.

**Fig. 2:**
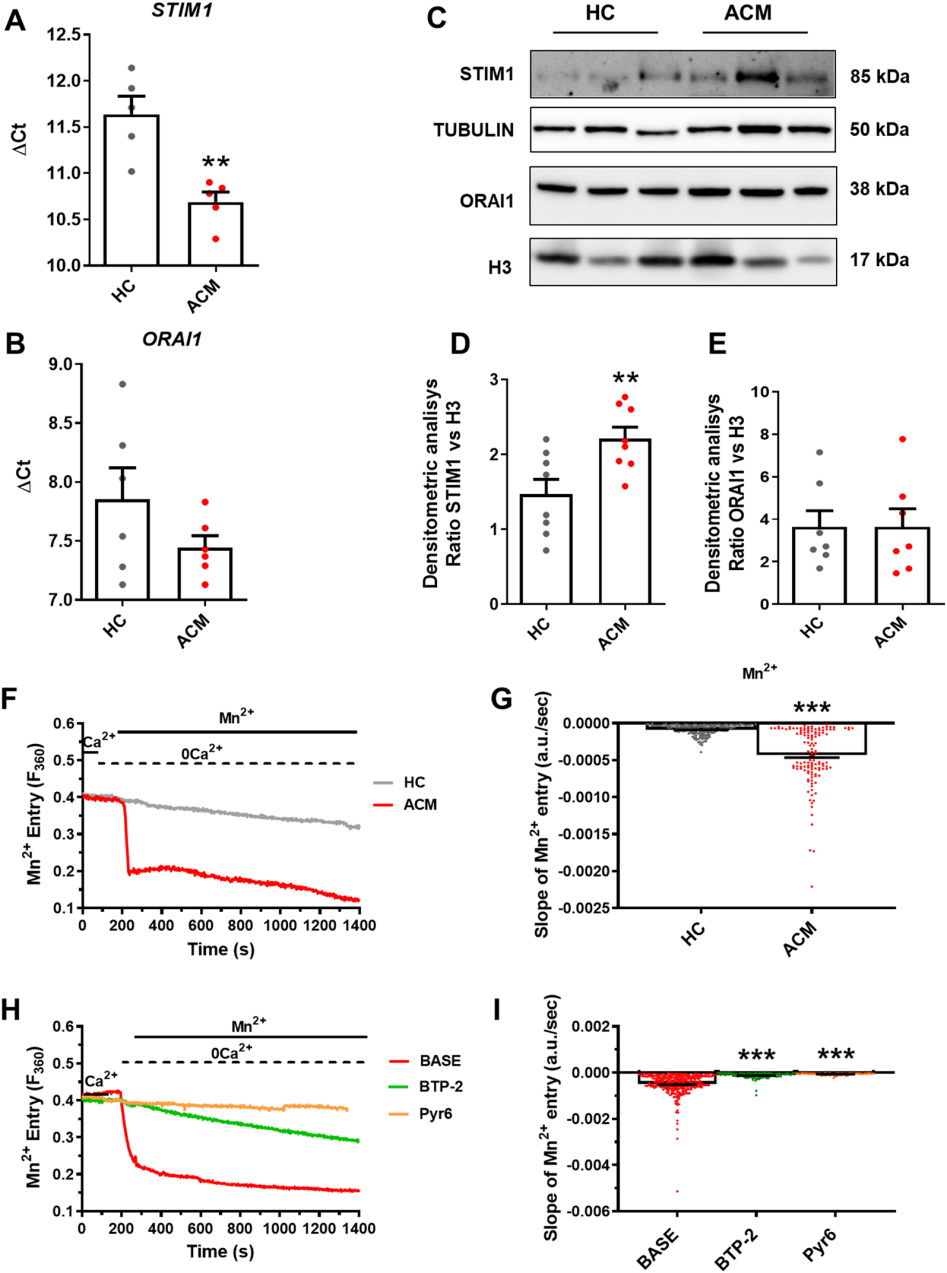
SOCE is constitutively active and enhanced in ACM C-MSC. Expression of *STIM1* (**A**) and *ORAI1* (**B**) in total RNA extracts of C-MSC from HC donors and ACM patients. GAPDH was used as a house-keeping gene and qRT-PCR data are presented as the genes threshold cycles (Ct) with respect to the housekeeping gene GAPDH (ΔCt) (n = 6 biological replicates; Two-tailed Student’s t-tests). **C** Representative images of WB analysis of proteins extracted from HC and ACM C-MSC cultured in GM, hybridized with anti-STIM1 and anti-ORAI1 antibodies. Immunostaining of the housekeeping H3 is shown for normalization. **D**–**E** Densitometric analysis of STIM1 (n = 8 biological replicates) and ORAI1 (n = 8 biological replicates) levels, normalized on H3 (Two-tailed Student’s t-tests). **F** Representative tracings of resting Ca^2+^ entry in C-MSC from HC donors and ACM patients evaluated by using the Mn^2+^-quenching technique. The measurements were performed on unstimulated cells cultured in GM. **(G)** Quenching rate of Fura-2/AM fluorescence induced by Mn^2+^ addition in unstimulated (BASE) C-MSC from HC donors and ACM patients (n = 182 cells HC *vs.* n = 129 ACM; Two-tailed Student’s t-tests). **H** Representative tracings of resting Ca^2+^ entry in C-MSC ACM patients evaluated by using the Mn^2+^-quenching technique. The measurements were performed on cells cultured in GM and maintained in the absence (BASE) of presence of BTP-2 (20 μM, 20 min) or PYR6 (10 μM, 10 min). **I** Quenching rate of Fura-2/AM fluorescence in unstimulated (BASE) or treated C-MSC from ACM patients (n = 280 cells BASE *vs.* n = 115 BTP-2 *vs.* n = 82 PYR6; one-way ANOVA test). Data information: mean ± SEM. *P < 0.05, **P < 0.01 and ***P < 0.0001

qRT-PCR and WB analysis revealed that STIM1 and ORAI1 transcripts and proteins were readily detectable in C-MSC (Fig. 2A, B). The comparison of ΔCt values showed that *STIM1* was significantly up-regulated in ACM C-MSC (*n* = 6; HC 11.64 ± 0.1969 *vs.* ACM 10.69 ± 0.1092; *P* = 0.0029; Fig. 2A) while there was no difference in the transcript levels of *ORAI1* (*n* = 6; HC 7.853 ± 0.2673 *vs.* ACM 7.443 ± 0.1008; *P* = 0.1818; Fig. 2B). These data were confirmed at protein levels by a WB analysis performed by using antibodies directed against STIM1 (*n* = 8; HC 1.473 ± 0.1957 *vs.* ACM 2.213 ± 0.1520; *P* = 0.0098; Fig. 2C, D) and ORAI1 (*n* = 8; HC 3.644 ± 0.7578 *vs.* ACM 3.643 ± 0.8494; *P* = 0.9995; Fig. 2C, E ).

Subsequently, we have explored the resting Ca^2+^ entry in HC and ACM C-MSC by using the Mn^2+^-quenching technique, to confirm that SOCE was constitutively active in ACM C-MSC. There was a rather linear quenching in Fura-2/AM fluorescence upon substitution of extracellular Ca^2+^ with Mn^2+^ in both HC and ACM cells (Fig. 2F, G). Furthermore, the rate of fluorescence decay was significantly higher in ACM C-MSC as compared to HC cells (*n* = 182; HC -8.552e-005 ± 5.157e-006 a.u./sec *vs*. *n* = 129; ACM -0.0004325 ± 3.415e-005 a.u./sec; *P* < 0.0001; Fig. 2F, G). Thus, both HC and ACM C-MSC display a basal Ca^2+^ permeability, but this is larger in the latter. Notably, constitutive Ca^2+^ entry was strongly inhibited by blocking ORAI1 with either PYR6 or BTP-2 (*n* = 280; BASE -0.0004924 ± 2.901e-005 a.u./sec *vs. n* = 115; BTP-2 -0.0001194 ± 1.199e-005 a.u./sec (*P* < 0.0001); *vs. n* = 82; PYR6 -5.723e-005 ± 4.416e-006 a.u./sec (*P* < 0.0001); Fig. 2H, I), thereby showing that the higher Ca^2+^ permeability in ACM C-MSC is due to a significant increase in constitutive SOCE.

In a separate set of experiments, we evaluated the expression CAV1.2, which mediates voltage-gated Ca^2+^ entry (VOGE) in cardiac myocytes. Both *CACNA1C* transcripts and proteins were down-regulated in ACM C-MSC compared to HC C-MSC (Additional file 1: Figure S9A-S9C). Accordingly, membrane depolarization with high-KCl solution induced a lower Ca^2+^ entry in ACM C-MSC (Additional file 1: Figure S9D-S9E), which was sensitive to the inhibition of CaV1.2 with nifedipine (Additional file 1: Figure S10). These observations are consistent with the minor role played by VOGE in spontaneous Ca^2+^ oscillations (Fig. 1A–E). Furthermore, addition of high-KCl solution did not resume the intracellular Ca^2+^ oscillations in ACM C-MSC in which the spontaneous Ca^2+^ activity ceased after a few Ca^2+^ spikes (data not shown).

### IP3-induced ER Ca^2+^ release is higher in ACM than in HC C-MSC

**Fig. 3:**
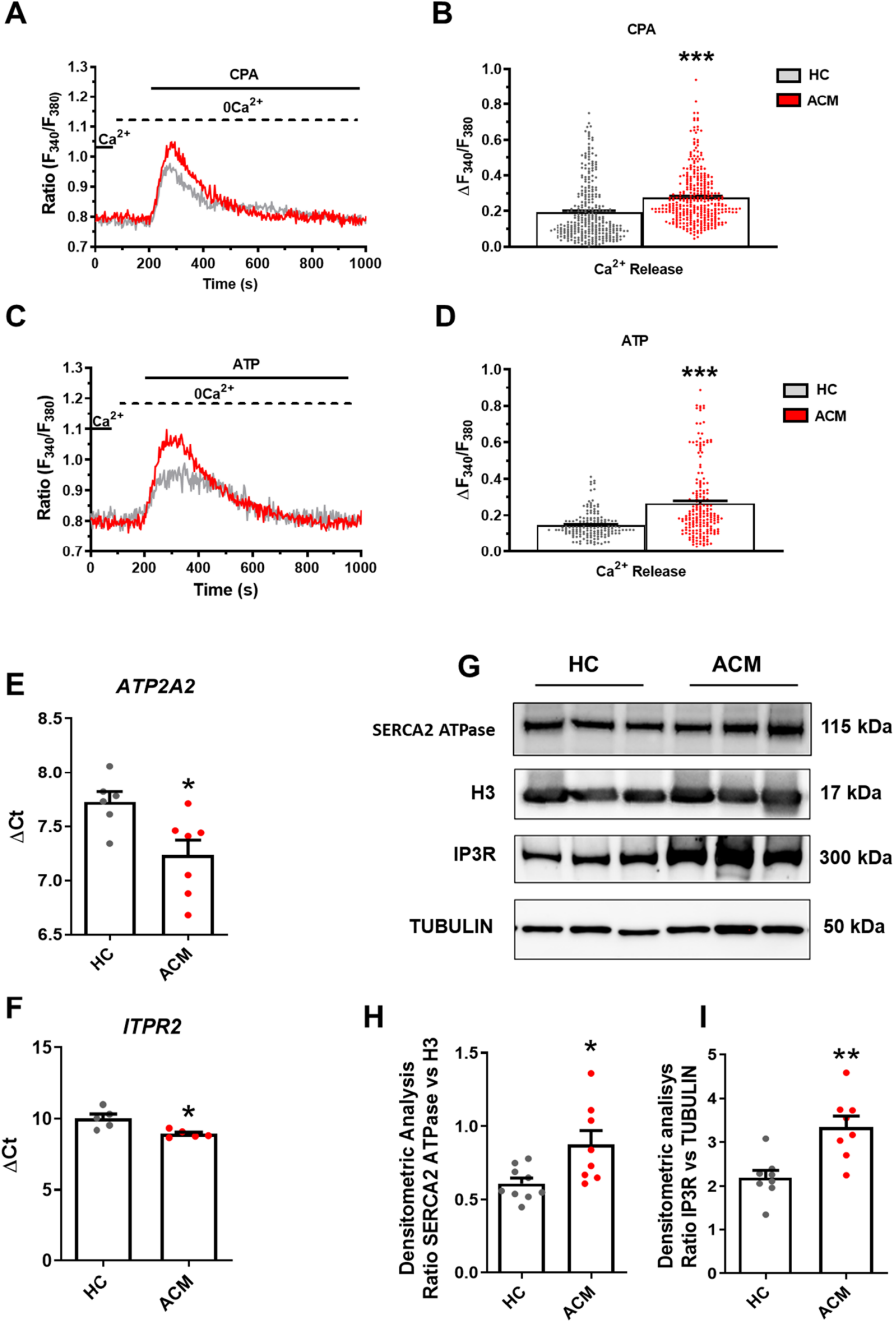
ER-dependent Ca^2+^-release through IP3Rs is larger in ACM C-MSC. **A** Representative tracings of the intracellular Ca^2+^ release evoked by CPA (30 μM) in C-MSC from HC donors and ACM patients. **B** Peak amplitude of CPA -evoked ER Ca^2+^ mobilization in HC and ACM C-MSC (n = 314 cells HC *vs.* n = 351 ACM; Two-tailed Student’s t-tests). **C** Representative tracings of the intracellular Ca^2+^ release evoked by ATP (100 μM) in C-MSC from HC donors and ACM patients. **D** Peak amplitude of ATP-evoked ER Ca^2+^ mobilization in HC and ACM C-MSC (n = 158 cells HC *vs.* n = 196 ACM Two-tailed Student’s t-tests). Expression of *ATP2A2* **E** and *ITPR2* **F** in total RNA extracts of C-MSC from HC donors and ACM patients. GAPDH was used as a house-keeping gene and qRT-PCR data are presented as the genes threshold cycles (Ct) with respect to the housekeeping gene GAPDH (ΔCt) (n = 6 biological replicates; Two-tailed Student’s t-tests). **G** Representative images of WB analysis of proteins extracted from HC and ACM C-MSC cultured in GM, hybridized with anti-SERCA2 ATPase and anti-IP3R antibodies. Immunostaining of the housekeeping H3 or Tubulin are shown for normalization. Densitometric analysis of SERCA2 ATPase (n = 8 biological replicates; **H**) and IP3R (n = 8 biological replicates; **I**) levels, normalized on H3 and Tubulin respectively (Two-tailed Student’s t-tests). Data information: mean ± SEM. *P < 0.05, **P < 0.01 and ***P < 0.0001

The larger resting Ca^2+^ entry in ACM C-MSC could result in an increase in the ER Ca^2+^ pool that is mobilized by IP3 during the spontaneous Ca^2+^ activity. Therefore, we directly evaluated the differences in the releasable ER Ca^2+^ by challenging HC and ACM C-MSC with CPA under 0Ca^2+^ conditions. CPA-induced ER Ca^2+^ release was significantly increased in ACM C-MSC as compared to HC C-MSC (*n* = 314; HC 0.1932 ± 0.009151 a.u. *vs. n* = 351; ACM 0.2767 ± 0.008012 a.u.; *P* < 0.0001; Fig. 3A, B). To further corroborate these data, we challenged the cells with the IP3-producing autacoid, ATP. Again, ATP-induced IP3-dependent ER Ca^2+^ release was significantly higher in ACM C-MSC (*n* = 158; HC 0.1463 ± 0.005810 a.u. *vs. n* = 196; ACM 0.2654 ± 0.01419 a.u.; *P* < 0.0001; Fig. 3C, D). Intriguingly, blocking the constitutive SOCE with either PYR6 or BTP-2 significantly inhibited both CPA- and IP3-induced Ca^2+^ mobilization (Additional file 1: Figure S11). These observations confirm that the increase in constitutive SOCE contributes to enhance IP3-dependent ER Ca^2+^ release in ACM C-MSC.

To extend this information at the molecular level, we investigated the expression of SERCA, which sequesters the Ca^2+^ entering the cell via the SOCE pathway, and the IP3R. Both *ATP2A2* (*n* = 8; HC 7.723 ± 0.09759 *vs.* ACM 7.233 ± 0.1395; *P* = 0.0178; Fig. 3E) and *ITPR2* (*n* = 6; HC 10.01 ± 0.3118 *vs.* ACM 8.932 ± 0.1204; *P* = 0.0120; Fig. 3F) transcripts were expressed at significantly higher levels in ACM C-MSC as compared to HC C-MSC. Densitometric analysis confirmed that both SERCA (*n* = 8; HC 0.6100 ± 0.03749 *vs.* ACM 0.8763 ± 0.09475; *P* = 0.0155; Fig. 3G, H) and IP3R (*n* = 8; HC 2.190 ± 0.1706 *vs.* ACM 3.345 ± 0.2540; *P* = 0.0020; Fig. 3G, I) proteins were expressed in both cell types and significantly higher in ACM than HC C-MSC. Furthermore, qRT-PCR and WB analysis (data not shown-no signal detected) indicated that phospholamban, known inhibitory cofactor for cardiac SERCA, is not expressed in C-MSC.

Thus, an increase in SERCA expression further enhances the ER Ca^2+^ load, whereas the up-regulation of IP3R contributes to boost IP3-induced Ca^2+^ release during spontaneous Ca^2+^ oscillations in ACM C-MSC.

### Flecainide blocks intracellular Ca^2+^ oscillations by blocking constitutive SOCE in ACM C-MSC

**Fig. 4:**
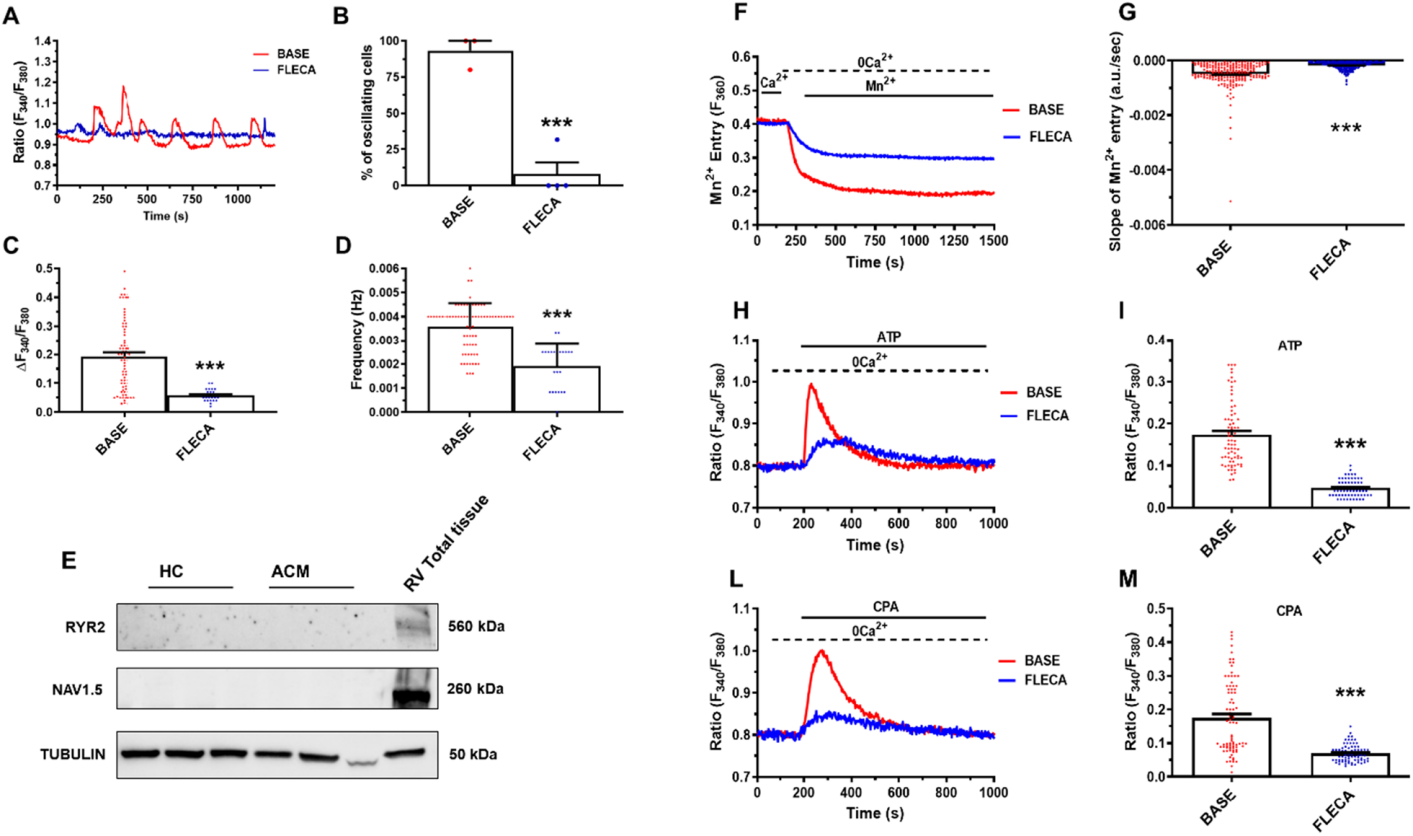
Flecainide inhibits spontaneous Ca^2+^ oscillations in ACM C-MSC release by inhibiting constitutive SOCE. **A** Representative Ca^2+^ traces in C-MSC from ACM patients loaded with Fura-2/AM. The measurements were performed on unstimulated cells cultured in GM in absence (BASE) or presence of flecainide (FLECA, 10 μM). **B**–**D** The graphs represent respectively: **B** percentage of oscillating cells **(**n = 76 cells BASE *vs.* FLECA n = 199 cells; Two-tailed Student’s t-tests**)**; **C** oscillation amplitude **(**n = 76 cells BASE *vs.* FLECA n = 22/199 cells; Two-tailed Student’s t-tests**)** and **D** oscillation frequency **(**n = 76 cells BASE *vs.* FLECA n = 22/199 cells; Two-tailed Student’s t-tests**)**. **E** Representative images of WB analysis of proteins extracted from HC and ACM C-MSC cultured in GM, hybridized with anti-RYR2 and anti-NAV1.5 antibodies. Immunostaining of the housekeeping Tubulin are shown for normalization. RV total lysate is loaded as positive control (n = 8 biological replicates). **F** Representative tracings of resting Ca^2+^ entry in C-MSC from ACM patients evaluated by using the Mn^2+^-quenching technique. The measurements were performed on cells cultured in GM in the absence (BASE) or presence of flecainide (FLECA, 10 μM, 30 min). **G** Basal rate of Fura-2/AM quenching in unstimulated or treated C-MSC (n = 280 cells BASE *vs.* n = 290 cells FLECA; Two-tailed Student’s t-tests). **H** Representative tracings of intracellular Ca^2+^ release evoked by ATP (100 µM) in the absence (BASE) and in the presence of flecainide (Fleca, 10 μM, 30 min). **I** Peak amplitude of ATP-evoked ER Ca^2+^ mobilization in the absence (BASE) and in the presence of flecainide (n = 76 cells BASE *vs.* n = 65/97 cells FLECA; Two-tailed Student’s t-tests). **L** Representative tracings of intracellular Ca^2+^ release evoked by CPA (30 µM) in the absence (BASE) and in the presence of flecainide (FLECA, 10 μM, 30 min). **M** Peak amplitude of CPA-evoked ER Ca^2+^ mobilization in the absence (BASE) and in the presence of flecainide (n = 86 cells BASE *vs.* n = 80/115 cells FLECA; Two-tailed Student’s t-tests). Data information: mean ± SEM. ***P < 0.001

Flecainide, an approved antiarrhythmic drug, may suppress the spontaneous SR Ca^2+^ release in mice and humans [42], thus effectively controlling triggered arrhythmias when used in diseases with SR Ca^2+^ impairment [43]. Consistently, flecainide (FLECA) reduced the percentage of oscillating cells (*n* = 76; BASE 93.33% ± 6.667 *vs. n* = 199; FLECA -7.950% ± 7.950; *P* = 0.0006; Fig. 4A, B), amplitude (*n* = 76; BASE 0.1933 ± 0.01399 a.u. vs. *n* = 22; FLECA 0.05818 ± 0.004682 a.u.; *P* < 0.0001; Fig. 4C) and frequency of Ca^2+^ oscillations in ACM C-MSC (*n* = 76; BASE 0.003589 ± 0.0001115 Hz *vs. n* = 22; FLECA 0.001920 ± 0.0001920 Hz; *P* < 0.0001; Fig. 4D). Flecainide exerts its anti-arrhythmic effect preferentially as an antagonist of the cardiac RYR2 and sodium (NAV1.5) channels [44–46]. However, qRT-PCR (data not shown-no signal detected) and WB (Fig. 4E) analysis indicated that RYR2 and NAV1.5 channels were not detectable either in HC C-MSC or ACM C-MSC. This finding was consistent with the evidence reported in Additional file 1: Figure S8D that caffeine did not induce any increase in [Ca^2+^]_i_ in C-MSC.

Based on these findings, we further explored the effect of flecainide on constitutive SOCE, which drives the IP3-dependent Ca^2+^ oscillations in ACM C-MSC. The Mn^2+^-quenching technique revealed that flecainide significantly reduced the rate of basal decay in Fura-2/AM fluorescence in ACM C-MSC (*n* = 217; BASE -0.0004877 ± 0.00003323 a.u./sec *vs. n* = 290; FLECA -0.0001713 ± 8.099e-006 a.u./sec; *P* < 0.0001; Fig. 4F–G), thereby mimicking the effect of PYR6 and BTP-2 (Fig. 2H–I).

We further validated the inhibitory effect of flecainide on SOCE by testing the impact on SOCE-mediated refilling of the ER Ca^2+^ pool. ACM C-MSC were subjected to ATP and CPA treatments in presence or absence of flecainide. Administration of either ATP (*n* = 76; 0.1739 ± 0.008547 a.u. *vs. n* = 65 out of 97; FLECA 0.04677 ± 0.002491 a.u.; *P* < 0.0001; Fig. 4H–I) or CPA (*n* = 86; 0.1751 ± 0.01163 a.u. *vs. n* = 80 out of 115; FLECA 0.06965 ± 0.002752 a.u.; *P* < 0.0001; Fig. 4L–M) resulted in an increase in [Ca^2+^]_i_ that was significantly reduced by flecainide treatment. These findings strongly suggest that flecainide could also exert a novel effect, targeted on constitutive SOCE, thereby interfering with the ER Ca^2+^ loading and abolishing spontaneous Ca^2+^ oscillations in ACM C-MSC.

### Spontaneous Ca^2+^ oscillations and CaMKII activity regulate adipogenesis and fibrosis in ACM C-MSC

**Fig. 5:**
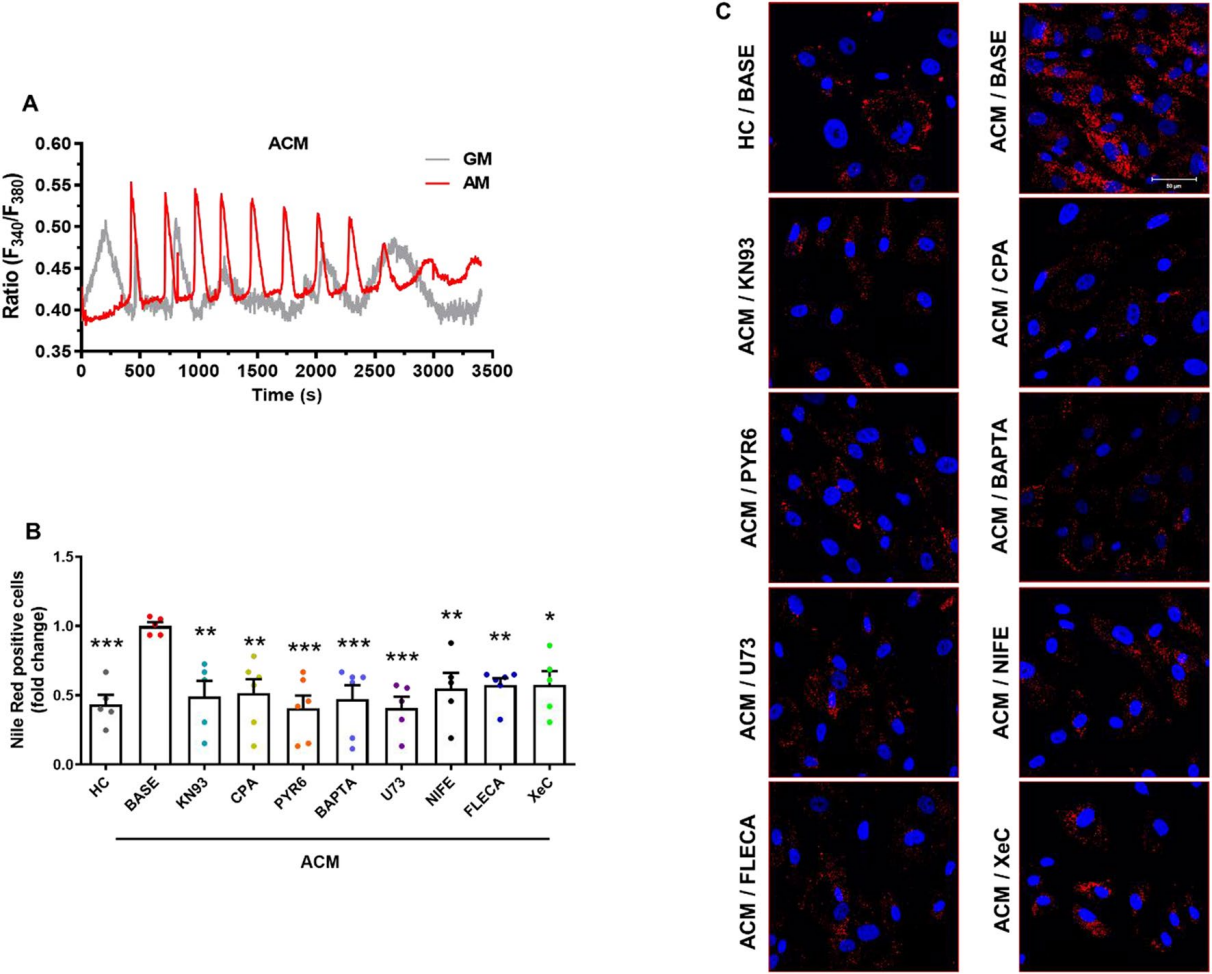
Ca^2+^ oscillations and CaMKII mediate ACM C-MSC adipogenic differentiation. **A** Representative Ca^2+^ traces in C-MSC from ACM patients loaded with Fura-2/AM. The measurements were performed on unstimulated cells cultured in GM or AM for 3 days. **B** Results of a FACS analysis of ACM C-MSC cultured in AM for 3 days supplemented or not with CaMKII inhibitor or [Ca^2+^]_i_ modulators (KN93, CPA, PYR6, BAPTA, U73, NIFE, FLECA 10 μM and XeC 1 μM; 3 days), and HC C-MSC, marked with Nile red. The mean fluorescence of Nile Red positive cells is shown for each condition relative to the values of basal level of ACM C-MSC in AM (BASE) (n = 5 biological replicates; one-way ANOVA test). **C** Representative images of Nile Red staining of ACM C-MSC cultured in AM for 3 days supplemented or not with [Ca^2+^]_i_ modulators or CaMKII inhibitor, and HC C-MSC. Nuclei are stained with Hoechst 33,342. Magnification is 40X and the scale bar indicates 50 μm. Data information: mean ± SEM. *P < 0.05, **P < 0.01 and ***P < 0.001

C-MSC are a source of adipocytes in ACM hearts and ACM-derived primary C-MSC are able to undergo adipogenic differentiation in vitro [11]. Therefore, we investigated whether changes in the oscillatory Ca^2+^ activity occur following induction of adipogenic differentiation. Recordings confirmed that spontaneous Ca^2+^ oscillations display a significantly higher amplitude and frequency in ACM C-MSC cultured in AM compared to the HC C-MSC in AM (Additional file 1: Figure S12A-S12C). Moreover, the amplitude and frequency of oscillations in both C-MSC types were increased following adipogenic stimulation compared to the growing culture conditions (Fig. 5A; Additional file 1: Figure S12A-S12C). We then explored the possibility to modulate the ACM adipogenic phenotype through two different approaches: by interfering with spontaneous Ca^2+^ oscillations or by acting specifically on CaMKII activation. We confirmed that pre-treating the cells with the membrane-permeant Ca^2+^ buffer, BAPTA [32], suppressed the intracellular Ca^2+^ oscillations in ACM C-MSC (Additional file 1: Figure S13). ACM C-MSC were cultured in AM in presence or absence of specific inhibitors. Following the treatments, we performed a Nile Red staining, marking neutral lipids (Fig. 5C). The percentage of Nile Red positive cells (Fig. 5B) were significantly reduced following the treatment with inhibitors that affect spontaneous Ca^2+^ release and likewise upon inhibition of CaMKII activity by KN93 treatment (*n* = 5; BASE 1.000 ± 0.02805 *vs.* HC 0.4351 ± 0.06891 (*P* = 0.0007); *vs.* KN93 0.4924 ± 0.1117 (*P* = 0.0026); *vs.* CPA 0.5153 ± 0.1001 (*P* = 0.0027); *vs.* PYR6 0.4071 ± 0.09161 (*P* = 0.0002); *vs.* BAPTA 0.4707 ± 0.1015 (*P* = 0.0009); *vs.* U73 0.4084 ± 0.08155 (*P* = 0.0004); *vs.* NIFE 0.5496 ± 0.1125 (*P* = 0.0092); *vs.* FLECA 0.5725 ± 0.05097 (*P* = 0.0100); *vs.* XeC 0.5763 ± 0.09776 (*P* = 0.0160); Fig. 5B). Notably, the lipid accumulation in ACM C-MSC following the treatments was comparable to HC C-MSC. Furthermore, all the treatments were able to modulate the expression of perilipin1 (PLIN1; Additional file 1: Figure S14).

**Fig. 6:**
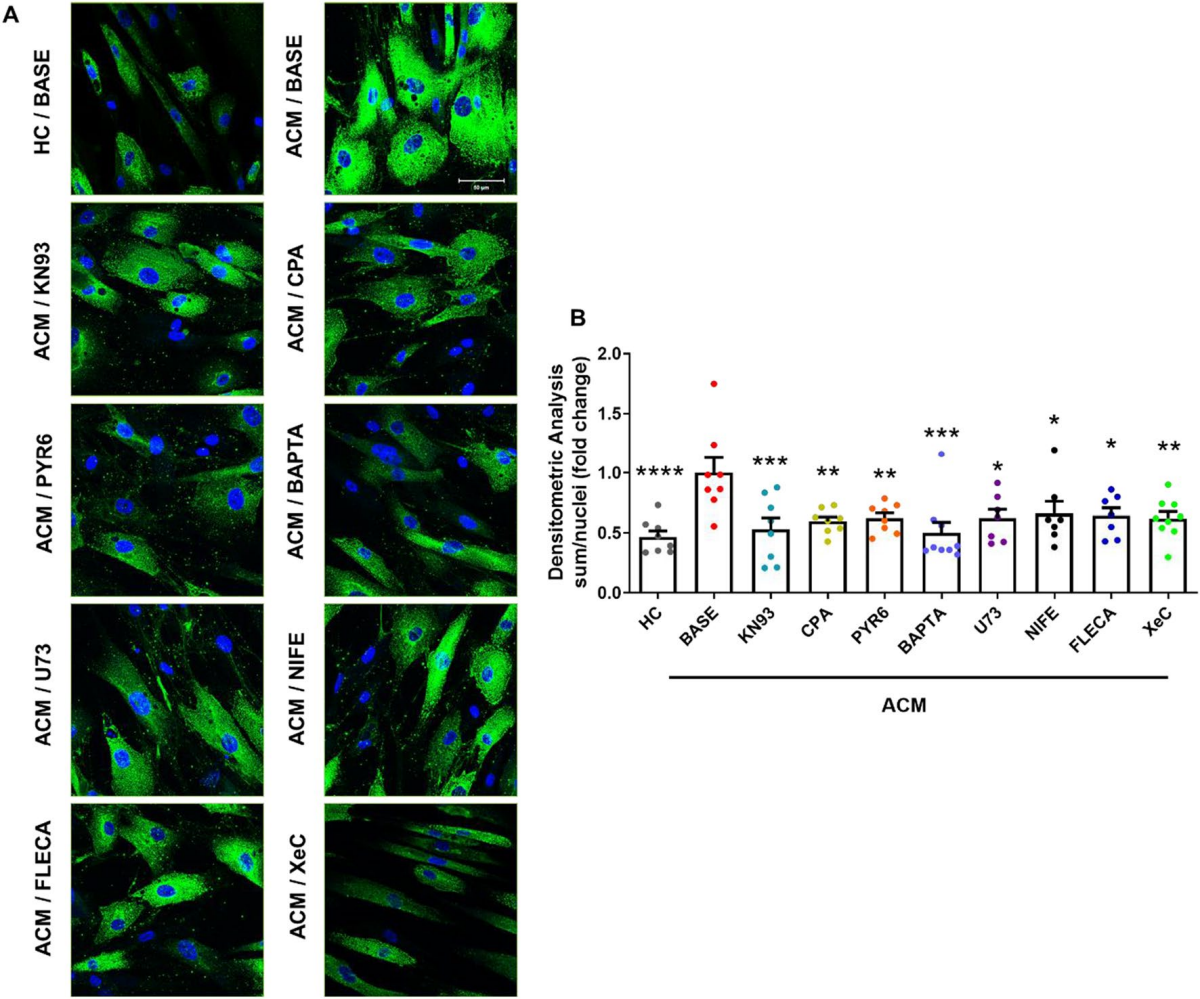
Ca^2+^ oscillations and CaMKII mediate ACM C-MSC fibrotic differentiation. **A** Representative images of Collagen staining of ACM C-MSC cultured in GM low serum (2% FBS) and TGF-β1 (5 ng/mL) for 3 days supplemented or with CaMKII inhibitor or [Ca^2+^]_i_ modulators (as in Fig. 5), and HC C-MSC. Nuclei are stained with Hoechst 33,342. Magnification is 40X and the scale bar indicates 50 μm. **B** Quantification of images of ACM C-MSC marked with Collagen. The mean fluorescence of Collagen is shown for each condition relative to the values of basal level of ACM C-MSC in in GM low serum (BASE) (n = 3 biological replicates; one-way ANOVA test). Data information: mean ± SEM. *P < 0.05, **P < 0.01 and ***P < 0.001.

Based on our previous data, demonstrating that C-MSC are a source of myofibroblasts in ACM heart [12], we tested the involvement of Ca^2+^ oscillations and CaMKII activity in ACM C-MSC mediated fibrosis. We analyzed collagen production in ACM C-MSC in pro-fibrotic medium cultures and treated with inhibitors of spontaneous Ca^2+^ oscillations and CaMKII activity (Fig. 6A–B). Immunofluorescence analysis demonstrated that the treatments reduced the production of collagen I (*n* = 3; BASE 1.000 ± 0.1272 *vs.* HC 0.4668 ± 0.04949 (*P* < 0.0001); *vs.* KN93 0.5290 ± 0.09522 (*P* = 0.0005); *vs.* CPA 0.5965 ± 0.03559 (*P* = 0.0039); *vs.* PYR6 0.6229 ± 0.04212 (*P* = 0.0081); *vs.* BAPTA 0.4986 ± 0.08911 (*P* = 0.0001); *vs.* U73 0.6201 ± 0.07373 (*P* = 0.0106); *vs.* NIFE 0.6610 ± 0.1009 (*P* = 0.0291); *vs.* FLECA 0.6415 ± 0.06574 (*P* = 0.0182); *vs.* XeC 0.6196 ± 0.05637 (*P* = 0.0055); Fig. 6B).

Notably, the modulation of ACM C-MSC fibro-adipose differentiation was reached both by known modulators of Ca^2+^ oscillations and by flecainide, which dampened the Ca^2+^ oscillations by blocking the constitutive SOCE (Fig. 5, 6).

### PKP2 expression affects the Ca^2+^ cycling machinery in C-MSC

Recently, it has been reported that *PKP2* is required for the transcription of genes that control intracellular Ca^2+^ cycling. Lack of *PKP2* parallels low expression of multiple molecules relevant to Ca^2+^ handling, leading to disruption of intracellular Ca^2+^ homeostasis in an ACM mouse model of cardiac Pkp2 deficiency [46].

By mimicking PKP2 haploinsufficiency, we evaluated the possible correlation between enhanced spontaneous Ca^2+^ oscillations in C-MSC and PKP2 expression.

To this purpose, we silenced *PKP2* in HC C-MSC, using lentiviral particles containing *PKP2* shRNA and we used as controls the same HC cells transduced with scramble construct.

**Fig. 7:**
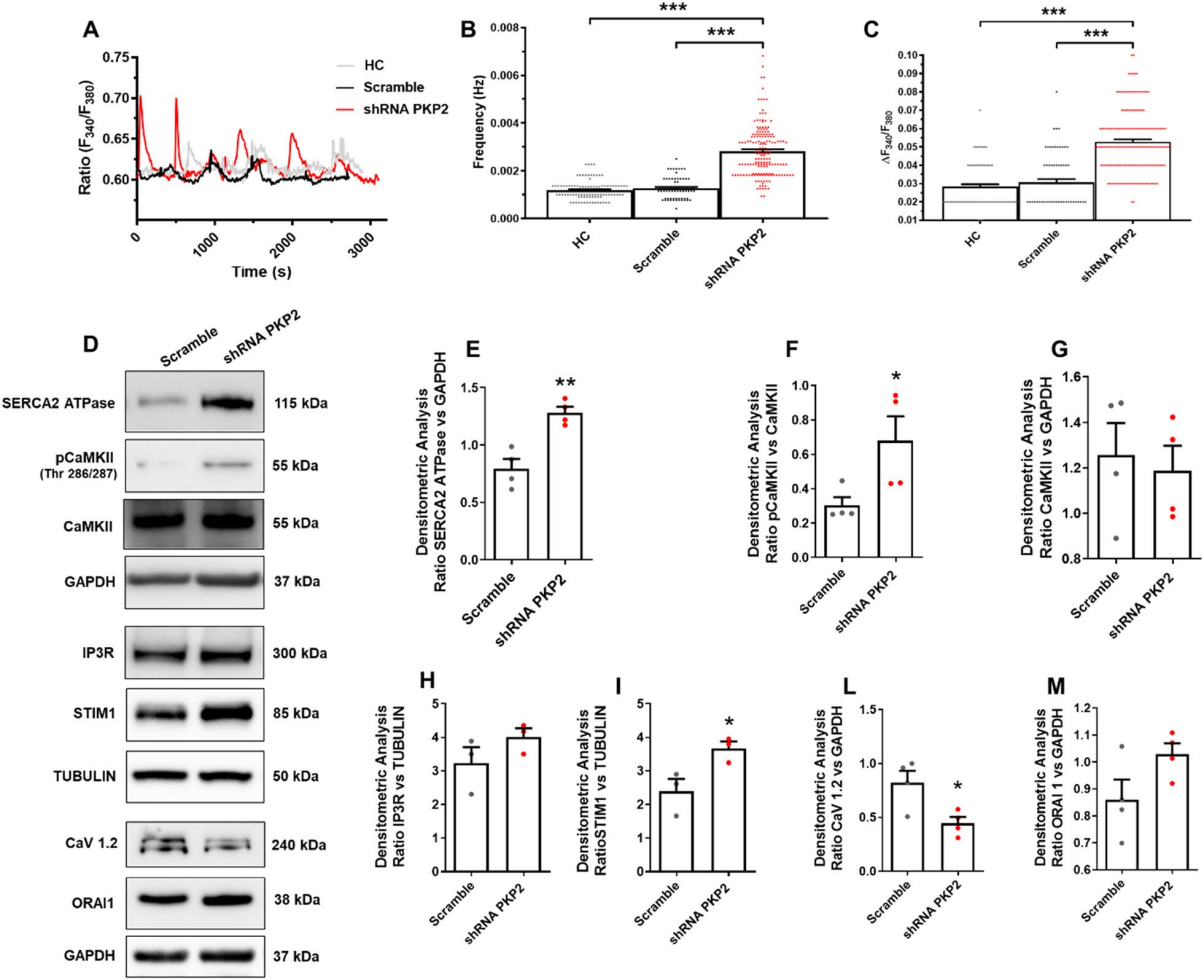
Study of the correlation between Ca^2+^ dysregulation and PKP2 expression. **A** Representative Ca^2+^ tracings in HC C-MSC treated with scramble shRNA or with shRNA selectively targeting PKP2 and loaded with Fura-2/AM. The measurements were performed on unstimulated cells cultured in GM. **B**–**C** The graphs represent respectively: **B** oscillation frequency **(**n = 93 out of 103 cells HC *vs.* n = 69 out of 92 cells Scramble *vs.* 170 out of 173 cells shRNA PKP2; One-Way Anova test**)** and **C** oscillation amplitude **(**n = 93 out of 103 cells HC *vs.* n = 69 out of 92 cells Scramble *vs.* 170 out of 173 cells shRNA PKP2; One-Way Anova test**)**. **D** Representative images of WB analysis of proteins extracted from HC C-MSC treated with shRNA scramble or PKP2 cultured in GM, hybridized with anti-SERCA2 ATPase, anti-pCaMKII, anti-CaMKII, anti-IP3R, anti-STIM1, anti-CAV1.2 and anti-ORAI1 antibodies. Immunostaining of the housekeeping GAPDH or Tubulin are shown for normalization. For each protein, the densitometric analysis were reported as follows: SERCA2 ATPase (**E**), pCaMKII (**F**), CaMKII (**G**), IP3R (**H**), STIM1 (**I**), CAV1.2 (**L**) and ORAI1 (**M**) (n = 4 biological replicates) levels, normalized on GAPDH or Tubulin (Two-tailed Student’s t-tests). Data information: mean ± SEM. *P < 0.05, **P < 0.01 and ***P < 0.0001

We confirmed *PKP2* silencing by a WB analysis (Additional file 1: Figure S15). Transduced C-MSC were loaded with Fura-2/AM to test if PKP2 deficiency affected intracellular Ca^2+^ oscillations. Lentiviral infection did not interfere with the oscillation profile given the comparable levels of HC C-MSC and Scramble infected HC C-MSC (Fig. 7A). Interestingly, HC C-MSC infected with shRNA *PKP2* display a significantly higher frequency (HC 0.001183 ± 4.426e-005 Hz, n = 103, *vs.* Scramble 0.001269 ± 5.400e-005, n = 92, Hz *P* < 0.0001; *vs. PKP2* shRNA 0.002816 ± 8.222e-005 Hz, n = 173, *P* < 0.0001; Fig. 7B) and amplitude (HC 0.02837 ± 0.001180 a.u., n = 103, *vs.* Scramble 0.03058 ± 0.001845, n = 92, a.u. *P* < 0.0001; *vs. PKP2* shRNA 0.05282 ± 0.001328; *P* < 0.0001; Fig. 7C) of Ca^2+^ oscillations compared to the HC C-MSC and scramble ones.

To further assess the correlation between PKP2 expression and the Ca^2+^ cycling machinery, we performed a WB analysis by using specific antibodies directed against SERCA, IP3R, STIM1, CAV1.2, and ORAI1. Densitometric analysis showed that both SERCA (*n* = 4; Scramble 0.7959 ± 0.08330 *vs. PKP2* shRNA 1.283 ± 0.05259; *P* = 0.0026; Fig. 7D–E) and STIM1 (*n* = 4; Scramble 2.390 ± 0.3758 *vs. PKP2* shRNA 3.665 ± 0.2155; *P* = 0.0422; Fig. 7D–I) proteins were significantly up-regulated in shRNA *PKP2* C-MSC while CAV1.2 is less expressed (*n* = 4; Scramble 0.8201 ± 0.1116 *vs. PKP2* shRNA 0.4450 ± 0.05746; *P* = 0.0243; Fig. 7D–L).

In addition, a slight, albeit not significant, increase of IP3R was observed in silenced cells (*n* = 4; Scramble 3.231 ± 0.4761 *vs. PKP2* shRNA 4.010 ± 0.2581; *P* = 0.2236; Fig. 7D–H). Of note, WB analysis of the phosphorylated form of CaMKII, demonstrated that *PKP2* silencing also resulted in increased CaMKII activity (*n* = 4; Scramble 0.3029 ± 0.04781 *vs. PKP2* shRNA 0.6787 ± 0.1425; *P* = 0.0466; Fig. 7D–F). There were no differences in ORAI1 (*n* = 4; Scramble 0.8595 ± 0.07435 *vs. PKP2* shRNA 1.028 ± 0.04090; *P* = 0.0944; Fig. 7D–M) and CaMKII (*n* = 4; Scramble 1.255 ± 0.1414 *vs. PKP2* shRNA 1.188 ± 0.1091; *P* = 0.7174; Fig. 7D–G) proteins.

Taken together, these findings confirm a correlation between the reduction in *PKP2* expression, as commonly occurs in ACM, and the dysregulation in Ca^2+^ handling machinery and CaMKII activity in human C-MSC.

## Discussion

Over the last few years, the idea that ACM is a disease not limited to the contractile component but instead a pathology that actively involves non-contractile cells become widely accepted [11, 12, 47–49]. Despite this, a full knowledge of the molecular players involved and a full understanding of the molecular mechanisms leading to ACM pathogenesis are still lacking.

Of note, common ACM triggers (such as adrenergic stimulation and mechanical stretch) regulate Ca^2+^-dependent processes, and crucial ACM phenotypes are determined by Ca^2+^-related processes, ranging from modulation of myocyte excitability and fate (apoptosis) to maturation/differentiation of stromal cells [34, 46, 47, 50–54].

**Fig. 8:**
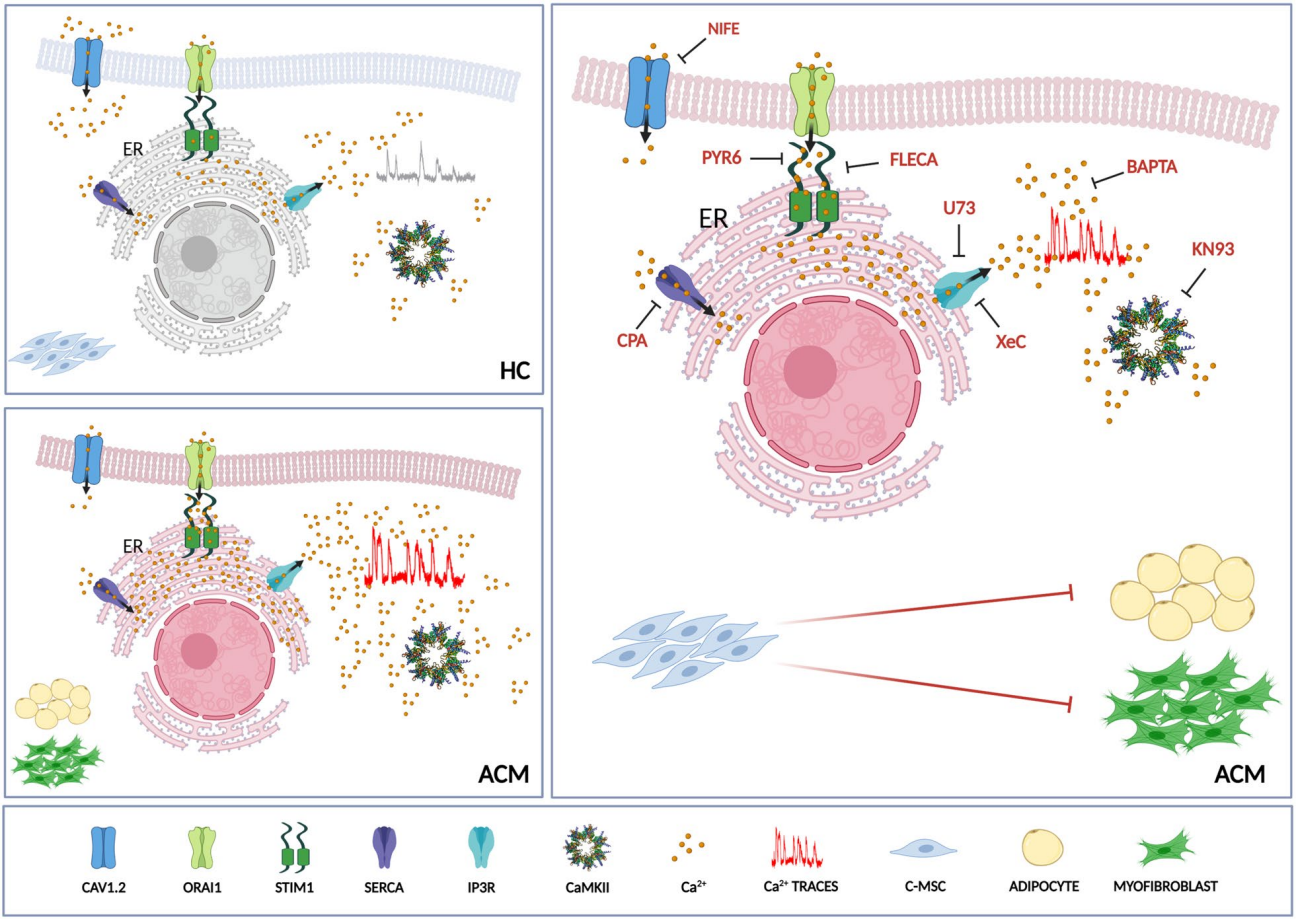
Characterization and targeting of Ca^2+^ imbalance and related phenotypes in ACM C-MSC. Left panel: Ca^2+^ handling machinery and CaMKII activity in HC (top) and ACM (bottom) C-MSC. Right panel: overview of approaches used to modulate fibro-fatty accumulation by targeting of Ca^2+^ oscillations and CaMKII activity in ACM C-MSC

Here we demonstrate for the first time that in ACM the Ca^2+^ handling machinery is dysregulated also in non-cardiomyocytes cells, i.e. in stromal cells, and that it contributes to the fibro-adipose degeneration process (Fig. 8). Our data, therefore, further expand the notion that Ca^2+^ dysregulation is a crucial determinant in the ACM phenotype [11, 12]. Intracellular Ca^2+^ signalling is instrumental to finely regulate proliferation and differentiation in human MSC isolated from multiple tissues [13, 17], but it is still unknown whether and how they modulate C-MSC fate.

The present study provides the first comprehensive characterization of the Ca^2+^ signalling machinery in human C-MSC and demonstrates that, in patients suffering from ACM, the Ca^2+^ toolkit and CaMKII undergo a complex dysregulation that ultimately results in stimulation of fibro-adipogenic differentiation. Indeed, our data show that spontaneous Ca^2+^ oscillations are present in cultured human C-MSC and occur in a significantly higher percentage and at enhanced amplitude and frequency in C-MSC isolated from ACM patients compared to what seen in HC. This, in turn, results in the hyperactivation of CaMKII, a pivotal effector of intracellular Ca^2+^ oscillations [23, 38].

Our results highlight the prevalence of *CAMK2G* and *CAMK2D* isoforms in human C-MSC, as it occurs in other cardiac cells [55]. Moreover, CaMKII activation was higher in ACM than in HC C-MSC. In agreement, an increase in Ca^2+^ oscillations can effectively boost CaMKII activation in ACM C-MSC, as shown by in vitro studies [23], cellular investigations [56], and in silico modelling [57]. The spontaneous Ca^2+^ oscillations finely tuning the differentiation outcome of bone marrow-derived MSC are shaped by rhythmic cycles of ER Ca^2+^ release through IP3R. Extracellular Ca^2+^ entry through SOCC is required to replenish the ER with Ca^2+^ in a SERCA-dependent manner, whilst VOGE is minimally contributory [14, 17]. Our data indicate that IP3R and SOCE are the major drivers of spontaneous Ca^2+^ oscillations also in ACM C-MSC. We provide the following evidences: i) the pharmacological blockade of SOCE with two selective ORAI1 inhibitors, i.e., PYR6 and BTP-2, reversibly prevented the spiking Ca^2+^ activity. Blocking VOGE through L-type CaV1.2 channels only weakly affected the Ca^2+^ transients. ii) spontaneous Ca^2+^ oscillations in ACM C-MSC were abrogated in most of the cells by blocking IP3R with the selective inhibitor, XeC, or by preventing the basal PLC-mediated production of IP3 with U73. Conversely, caffeine failed to mobilize intracellular Ca^2+^, since RYR2 was not expressed in ACM C-MSC. iii) spontaneous Ca^2+^ oscillations were suppressed by preventing Ca^2+^ reuptake into the ER with CPA, a selective inhibitor of SERCA activity, which further causes depletion of the ER Ca^2+^ store. Therefore, we can conclude that each Ca^2+^ transient is initiated by ER Ca^2+^ release through IP3R followed by ER Ca^2+^ refilling via SERCA. SOCE activation is required to maintain, and possibly trigger, the intracellular oscillations and downstream Ca^2+^-dependent signalling pathways.

The robust increase in spontaneous Ca^2+^ oscillations prompted us to hypothesize that the Ca^2+^ handling machinery was remodelled in ACM C-MSC. We found that SOCE was constitutively activated and resulted in a larger background Ca^2+^ entry in ACM as compared to HC C-MSC. In accordance, STIM1 and ORAI1 were both expressed in C-MSC but a significantly higher expression of STIM1 was found in ACM compared to the HC C-MSC. Although STIM2 is commonly regarded as the primary regulator of basal Ca^2+^ entry [58], constitutive activation of STIM1 enhances background Ca^2+^ influx and, thereby, increases resting [Ca^2+^]_i_ and/or ER Ca^2+^ levels in several genetic disorders. In agreement, both basal Ca^2+^ levels and the ER Ca^2+^ load are enhanced in ACM as compared to HC C-MSC [21, 22]. Similar to ACM C-MSC, adult CM isolated from transgenic mice overexpressing STIM1, displayed spontaneous Ca^2+^ oscillations and enhanced resting, i.e., diastolic [Ca^2+^]_i_ [59].

The increase in constitutive SOCE could per se lead to an increase in ER Ca^2+^ load in ACM C-MSC. In accordance, CPA-evoked intracellular Ca^2+^ release was also remarkably increased in ACM as compared to HC C-MSC. Furthermore, the pharmacological blockade of SOCE hampered both CPA- and ATP-induced endogenous Ca^2+^ mobilization from the ER, thereby confirming that the increase in basal Ca^2+^ permeability makes ACM C-MSC eager to release more Ca^2+^ during each IP3R-mediated Ca^2+^ transient. In addition, both SERCA and IP3R proteins are up-regulated, which, respectively, further increase ER Ca^2+^ loading and the ER Ca^2+^ releasing ability in patients-derived cells. Conversely, CaV1.2 is down-regulated and less active in ACM as compared to HC C-MSC, and, therefore, plays a minor role in spontaneous Ca^2+^ oscillations.

Arrhythmic events occurring in cardiomyopathies and channelopathies may be mostly associated to Ca^2+^ dysregulation in CM [60]. Research on human iPSC-CM revealed that Ca^2+^ imbalance seems mainly attributable to plasma membrane Ca^2+^ channels and transporters alterations. Changes in the action potential duration observed in *DSG2* iPSC-CM were demonstrated to be sustained by altered Na^+^/Ca^2+^ exchanger (NCX) currents [61]. A *PKP2*-mutated ACM model of hiPSC-CM showed slower cytoplasmic [Ca^2+^]_i_ relaxation with a prolonged relaxation time constant [62]. Electrophysiological studies in obscurin-mutated hiPSC-CM, highlighted that L-type Ca^2+^ current (ICaL) is increased in ACM- hiPSC-CM and this imbalanced ICaL may be linked to arrhythmic state [63]. In the CM context, flecainide administration represents an efficient strategy, based on its ability to suppress the spontaneous SR Ca^2+^ release in mice and humans, thus exhibiting an antiarrhythmic effect [43, 46]. For instance, flecainide prevents arrhythmias in a mouse model of Catecholaminergic Polymorphic Ventricular Tachycardia (CPVT) by inhibiting cardiac RYR2–mediated Ca^2+^ release [43, 64, 65] and is now considered a valid therapeutic approach in CPVT patients non-responsive to betablockade alone. The efficacy of flecainide has also been proven in ACM models. Data from a CM-specific tamoxifen-activated Pkp2 knock-out mouse (Pkp2-cKO) model demonstrate that flecainide treatment abolishes adrenergic arrhythmias [46]. Moreover, it is able to normalise spontaneous Ca^2+^ transients in hiPSC-CM with missense mutation in the *DSC2* gene [66]. Despite the historic hesitation in using class IC anti-arrhythmic in patients with structural heart disease [67], different recent evidence supported safety in the use of Flecainide in ACM [42, 68–70]. Currently, a pilot double-blind cross-over clinical trial (ClinicalTrial.Gov NCT03685149) is evaluating the effectiveness of flecainide for arrhythmic burden reduction in ACM patients. Our data demonstrated that flecainide reduced spontaneous Ca^2+^ oscillations in ACM C-MSC. Flecainide is known as a NaV1.5 and RYR2 blocker [44, 45], which are absent in C-MSC. Here we show for the first time that flecainide inhibits constitutive SOCE, thereby preventing ER Ca^2+^ refilling and interfering with rhythmical ER Ca^2+^ release through IP3R. Therefore, besides interfering with the Ca^2+^ handling machinery in CM, flecainide exerts an etiological beneficial effect on the stromal component of the ACM heart. The main pharmacological therapy currently recommended in the clinical practice is the use of β-blockers and eventually amiodarone [71]. These drugs have either direct or indirect effect on Ca^2+^ content and channel activity. However, to date, specific studies on their effect on ACM C-MSC are not available.

The ACM heart typically undergoes progressive replacement of the ventricular wall by fibrotic and/or adipose tissue resulting in impaired contractile function and providing a nonconductive substrate, a source for re-entrant arrhythmias [72]. Previous studies have highlighted that C-MSC contribute both to adipogenic remodelling and myocardial fibrosis in ACM hearts [11, 12]. Specifically, C-MSC exposed to a pathological microenvironment (i.e. determined by an excess of TGFβ and oxidized-LDL) contribute to induce collagen and fat accumulation [12, 48]. The induction of differentiation programs in MSC involves an alteration of spontaneous Ca^2+^ oscillations [13, 14] whose frequency can either increase or diminish depending on the differentiation outcome. Herein, we observed a significant increase in the oscillation frequency in C-MSC undergoing adipogenic differentiation that was further enhanced in patients-derived cells. Lipid accumulation and collagen production in ACM C-MSC is reduced by the pharmacological blockade of CaMKII with KN-93, which suggests that the oscillatory Ca^2+^ signal in these cells increases during adipogenesis to support CaMKII activation. Of note, CaMKII has been shown to promote adipogenesis in porcine BM-MSC by stimulating the PI3K/Akt-Fox01 signalling pathway to increase the expression of two crucial adipogenic transcription factors [29], such as peroxisome proliferator activated receptor γ (PPARγ) and CCAAT/ enhancer binding protein α (C/EBPα) [73]. In addition, the inhibition of CaMKII is able to reduce cardiac fibroblast proliferation, the secretion of TGF-β1 and TNF-α, and can revert the upregulation of MMPs and collagen, confirming its involvement in extracellular matrix regulation [74]. Of note, CaMKII activity interferes the angiotensin II-mediated differentiation of cardiac fibroblasts into myofibroblasts by inducing collagen deposition thus contributing to cardiac remodelling [75].

Remarkably, we obtained substantial results by either using compounds acting on known SOCE targets or flecainide that regulated SOCE activity in our cells.

We finally evaluated the possible role of PKP2 in the remodelling of the Ca^2+^ toolkit observed in ACM C-MSC [76], based on the recent reports highlighting a link between PKP2 and the control of the Ca^2+^ cycling machinery in CM [46]. Specifically, by using the Pkp2-cKO mouse model, Cerrone and co-workers found that several components of Ca^2+^ handling machinery were down-regulated in CM, causing a reduction in SR Ca^2+^ leakage, thereby leading to an increase in SR Ca^2+^ content [46]. Similar to ACM C-MSC, but through a different mechanism (e.g., RYR2 *vs.* IP3R), the enhanced SR Ca^2+^ load results in an increase in amplitude and frequency of spontaneous Ca^2+^ sparks in mouse Pkp2-cKO CM [46]. The relationship between PKP2 and the Ca^2+^ machinery in CM was also pointed out by a bioinformatics approach, based on transcriptomic information from human hearts, that confirmed that at lower expression of *PKP2* corresponds to a lower expression of the genes encoding for RYR2, Ankyrin-B and CAV1.2 [5]. Interestingly, our data, obtained by mimicking PKP2 haploinsufficiency in C-MSC, demonstrated that the alterations in the intracellular Ca^2+^ cycling in ACM C-MSC and CaMKII activation can be linked to PKP2 levels in the cells. Indeed, both spontaneous Ca^2+^ activity and CaMKII activation were remarkably enhanced in HC C-MSC infected with shRNA *PKP2*. Moreover, as observed in ACM C-MSC, genetic downregulation of PKP2 led to an increase in the expression levels of STIM1 and SERCA proteins, and in the reduction of CAV1.2 protein. However, our experiments included C-MSC collected from a pool of ACM patients, not necessarily bearing *PKP2* mutations, which suggests that increased Ca^2+^ load and Ca^2+^ homeostasis dysregulation could be a common mechanism in the setting of different genetic forms of the disease. As an example, data on *DSG2*-mutated CM also showed Ca^2+^ imbalance and increased SR load [61, 77].

## Conclusions

In conclusion, our results provide evidence that Ca^2+^ imbalance in ACM extends beyond the CM compartment and involves stromal cardiac cells and the fibro-adipose degeneration process (Fig. 8). Moreover, we provided evidence about a novel mode of action of flecainide in ACM, which is effective in attenuating C-MSC fibro-fatty differentiation through the newly described molecular target SOCE. Our results add further rationale about the use of flecainide as pharmacological treatment of ACM and await efficacy confirmation on cardiac remodelling in a clinical trial in ACM patients.

## Supplementary Information

**Additional file 1**: **Text**. IP3-induced intracellular Ca^2+^ release and SOCE drive spontaneous Ca^2+^ oscillations in ACM C-MSC. **Fig. S1**. Resting [Ca^2+^]_i_in C-MSC. **Fig. S2**. Suramin and MRS-2179 inhibit the spontaneous Ca^2+^ activity in ACM C-MSC. **Fig. S3**. CaMKII expression in human-derived C-MSC. **Figure S4**: SOCE and IP3Rs drive the spontaneous Ca^2+^ oscillations in ACM C-MSC. **Fig. S5.** Effect of XeC on spontaneous Ca^2+^ oscillations in ACM C-MSC. **Fig. S6**. Contribution of VOCC and reverse-mode NCX to spontaneous Ca^2+^ oscillations in ACM C-MSC. **Fig. S7.** IP3R, but not RYR, contribute to spontaneous Ca^2+^ oscillations in ACM C-MSC. **Fig. S8**. Ryanodine do not affect spontaneous Ca^2+^ oscillations in ACM C-MSC. **Fig. S9**. Voltage-Gated Ca^2+^ Entry is lower in ACM C-MSC. **Fig. S10.** Nifedipine inhibits Ca^2+^ response to High K^+^. **Fig. S11**. Blocking constitutive SOCE prevents ER Ca^2+^ release in ACM C-MSC. **Fig. S12.** Spontaneous Ca^2+^ oscillations during adipogenesis. **Fig. S13**. BAPTA inhibit the spontaneous Ca^2+^ activity of ACM C-MSC. **Fig. S14**. PLIN1 modulation in ACM C-MSC. **Fig. S15.** PKP2 silencing in C-MSC. **Table S1**. Clinical data of ACM patients enrolled for biopsy samples. **Table S2.** Clinical features of the deceased tissue donors (with healthy heart) enrolled in this study. **Table S3.** Primer sequences 5’ - 3’. **Table S4.** Primary antibodies

## Data Availability

The data supporting the findings of this study are available within the article and its supplementary materials. All other supporting data are available from the corresponding author on reasonable request.

## Abbreviations

ACM: Arrhythmogenic cardiomyopathy
PKP2: Plakophilin2
JUP: Plakoglobin
DSP: Desmoplakin
DSG2: Desmoglein-2
DSC2: Desmocollin-2
CM: Cardiomyocytes
C-MSC: Cardiac Mesenchymal Stromal Cells
Ca^2+^: Calcium
ER: Endoplasmic reticulum
IP3R: Inositol-1,4,5-trisphosphate receptors
SOCC: Store-operated Ca^2+^ channels
PM: Plasma membrane
SERCA: Sarco-Endoplasmic Reticulum Ca^2+^-ATPase
STIM: Stromal interaction molecule
ORAI: Calcium release-activated calcium channel protein
VOCC: L-type voltage-operated Ca^2+^ channels
SOCE: Store-operated Ca^2+^ entry
CaMKII: Ca^2+^/Calmodulin-dependent protein kinase II
SR: Sarcoplasmic Reticulum
HC: Healthy control
GM: Growing medium
AM: Adipogenic medium
TGF-β1: Transforming growth factor-beta
CPA: Cyclopiazonic Acid
VOGE: Voltage-gated Ca^2+^ entry
RYR2: Ryanodine Receptor 2
XeC: Xestospongin C
PLIN1: Perilipin1
