# Supplementary material for: Ca2+ dysregulation in cardiac stromal cells sustains fibro-adipose remodeling in Arrhythmogenic Cardiomyopathy and can be modulated by flecainide
Source: J Transl Med. 2022 Nov 12;20:522. doi: 10.1186/s12967-022-03742-8 (PMC9652790; doi:10.1186/s12967-022-03742-8)
Supplement: Supplementary file 1 — Additional file 1: Text. IP3-induced intracellular Ca2+ release and SOCE drive spontaneous Ca2+ oscillations in ACM C-MSC. Fig. S1. Resting [Ca2+]iin C-MSC. Fig. S2. Suramin and MRS-2179 inhibit the spontaneous Ca2+ activity in ACM C-MSC. Fig. S3. CaMKII expression in human-derived C-MSC. Figure S4: SOCE and IP3Rs drive the spontaneous Ca2+ oscillations in ACM C-MSC. Fig. S5. Effect of XeC on spontaneous Ca2+ oscillations in ACM C-MSC. Fig. S6. Contribution of VOCC and reverse-mode NCX to spontaneous Ca2+ oscillations in ACM C-MSC. Fig. S7. IP3R, but not RYR, contribute to spontaneous Ca2+ oscillations in ACM C-MSC. Fig. S8. Ryanodine do not affect spontaneous Ca2+ oscillations in ACM C-MSC. Fig. S9. Voltage-Gated Ca2+ Entry is lower in ACM C-MSC. Fig. S10. Nifedipine inhibits Ca2+ response to High K+. Fig. S11. Blocking constitutive SOCE prevents ER Ca2+ release in ACM C-MSC. Fig. S12. Spontaneous Ca2+ oscillations during adipogenesis. Fig. S13. BAPTA inhibit the spontaneous Ca2+ activity of ACM C-MSC. Fig. S14. PLIN1 modulation in ACM C-MSC. Fig. S15. PKP2 silencing in C-MSC. Table S1. Clinical data of ACM patients enrolled for biopsy samples. Table S2. Clinical features of the deceased tissue donors (with healthy heart) enrolled in this study. Table S3. Primer sequences 5’ - 3’. Table S4. Primary antibodies [file 12967_2022_3742_MOESM1_ESM.docx]

**Ca^2+^ dysregulation in cardiac stromal cells sustains fibro-adipose remodeling in Arrhythmogenic Cardiomyopathy and can be modulated by flecainide**

**Supplementary Material**

**Supplementary text**

**IP3-induced intracellular Ca^2+^ release and SOCE drive spontaneous Ca^2+^ oscillations in ACM C-MSC**

We explored the sources of the spiking Ca^2+^ activity. Removal of extracellular Ca^2+^ (0Ca^2+^) during ongoing oscillations caused an abrupt interruption of intracellular Ca^2+^ transients, which resumed upon restitution of external Ca^2+^ to the perfusate (*n*=491; ACM Ca^2+^ 95.02%+3.942 *vs.* ACM in 0Ca^2+^ no response; **Figure S4A- S~~4~~B**). To further define the Ca^2+^ entry pathway(s) that maintain the spontaneous Ca^2+^ activity in ACM-MSC, we pretreated the cells with PYR6 and BTP-2, which inhibit SOCE by selectively targeting ORAI1 (1, 2). As shown in **Figure S4C** and summarized in **Figure S4D** (*n*=100; BASE 94%+3.480 *vs.* *n*=155; Pyr6 no response; *vs.* *n*=162; BTP-2 no response), both drugs reversibly inhibited spontaneous Ca^2+^ oscillations in ACM C-MSC. Nifedipine, a selective inhibitor of L-type VOCC (3), only slightly affected the amplitude of repetitive Ca^2+^ transients (**Figure S7A-S7C**), while KB-R7943 (KB-R), which interferes with the reverse-mode of NCX, did not significantly affect intracellular Ca^2+^ oscillations (**Figure S7D-S7F**). Therefore, our data show that SOCE represents the main pathway for Ca^2+^ entry, facilitating enhanced Ca^2+^ activity in ACM C-MSC.

In agreement with these observations, spontaneous Ca^2+^ oscillations were abolished by (i) cyclopiazonic acid (CPA); (*n*=143; BASE 91.60%+5.305 *vs*. *n*=165; CPA no response; **Figures S4E- S4F**), which depletes the ER Ca^2+^ store by inhibiting SERCA activity; (ii) U73122 (U73), which inhibits the IP3-producing enzyme, phospholipase C (PLC), thereby preventing IP3 production (*n*=92; BASE 96.40%+3.600 *vs*. *n*=165; U73 no response; **Figures S4G- S4H**); and (iii) Xestospongin C (2) (XeC), which selectively blocks IP3Rs (**Figure S4I- S4M**). In a fraction of ACM C-MSC, XeC did not suppress the spontaneous Ca^2+^ activity (*n*=76; BASE 96.67%+3.333 *vs*. *n*=144; XeC 24.28%+24.28; *P=*0.0539; **Figure S4M**), but significantly reduced the amplitude and frequency of repetitive Ca^2+^ transients (**Figure S5A-S5B**). To further confirm the role of IP3 in shaping the spontaneous Ca^2+^ oscillations, we used ATP, which is known to increase IP3 production (4). Preliminary experiments confirmed that ATP stimulates PLC to induce IP3-mediated Ca^2+^ release from the ER also in C-MSC (**Figure S8A-S8B**). The addition of ATP resumed the intracellular Ca^2+^ oscillations in ACM C-MSC in which the spontaneous Ca^2+^ activity ceased after a few Ca^2+^ spikes (**Figure S8C**). Conversely, caffeine, a selective agonist of RYRs, failed to mobilize intracellular Ca^2+^ in C-MSC (**Figure S8D**). In agreement with this finding, the pharmacological blockade of RyRs with the plant alkaloid ryanodine did not affect the spontaneous Ca^2+^ oscillations (**Figure S8**).

**Supplementary figures and tables**

**Fig. S1. Resting [Ca^2+^]_i_ in C-MSC.**

Resting [Ca^2+^]_i_ measurements in C-MSC from HC donors and ACM patients. The cells were loaded with the Ca^2+^-sensitive fluorophore, Fura-2/AM (2 μM, 30 min at 37°C), and the basal [Ca^2+^]_i_ was calibrated by following the Grynkiewicz method. The measurements were performed on unstimulated cells cultured in GM. (HC 77.85+6.074 nM, n=171, *vs.* ACM 169.3+20.62 nM, n=107; *P* < 0.0001; Two-tailed Student’s t-tests).

Data information: mean+SEM. ****P* < 0.0001.

**
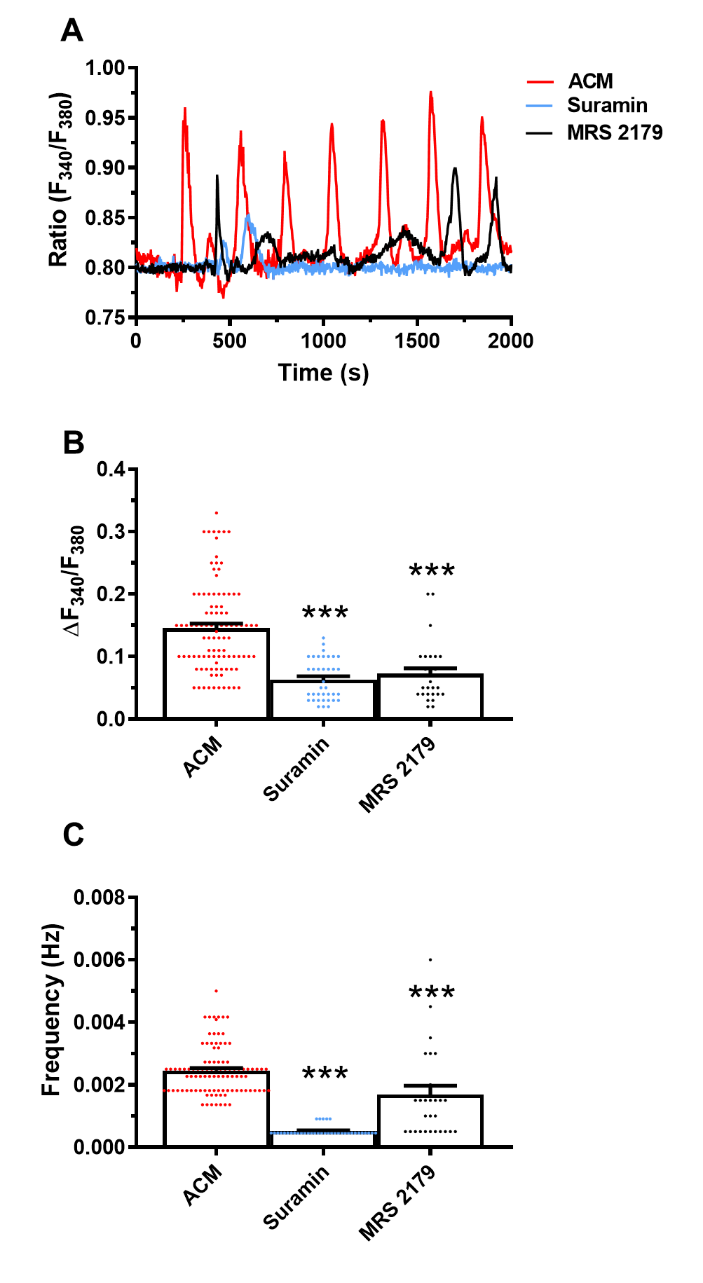
**

**Fig. S2. Suramin and MRS-2179 inhibit the spontaneous Ca^2+^ activity in ACM C-MSC.**

**(A)** Representative Ca^2+^ traces in C-MSC from ACM patients loaded with Fura-2/AM. The measurements were performed on cells cultured in GM in absence or presence of Suramin (10 µM) or MRS 2179 (20 µM). **(B-C)** Quantification of the spontaneous Ca^2+^ oscillations recorded in the absence and presence of P2Y receptor inhibitors. The graphs represent respectively: (**B)** the oscillation amplitude **(**ACM 0.1456+0.007073 a.u., n=100, *vs.* Suramin 0.06297+0.005395 a.u., n=37 out of 150 cells, vs. MRS 2179 0.07241+0.008605 a.u., n=29 out of 131 cells; P< 0.0001**)**; **(C)** oscillation frequency **(**ACM 0.002455+7.844e-005 a.u., n=100, *vs.* Suramin 0.0005160+2.590e-005 a.u., n=37 out of 150 cells, vs. MRS 2179 0.001690+0.0002790 a.u., n=29 out of 131 cells; P< 0.0001**).**

Data information: mean+SEM. ****P* < 0.0001.


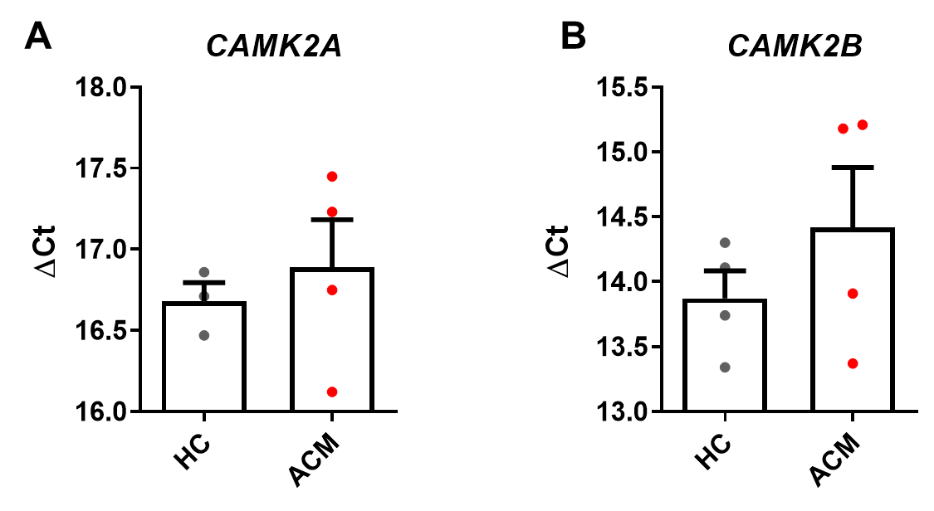


Fig. S3. CaMKII expression in human-derived C-MSC.

Expression of *CAMK2A* (n=4; HC 16.68+0.1136 *vs.* ACM 16.89+0.2946; P=0.5907; Two-tailed Student’s t-tests); (**A**) and *CAMK2B* (n=4; HC 13.87+0.2122 *vs.* ACM 14.42+0.4623; P=0.3251; Two-tailed Student’s t-tests); (**B**) isoforms in total RNA extracts of C-MSC from HC donors and ACM patients. GAPDH was used as a house-keeping gene and qRT-PCR data are presented as the genes threshold cycles (Ct) with respect to the housekeeping gene GAPDH (ΔCt).

Data information: mean+SEM.


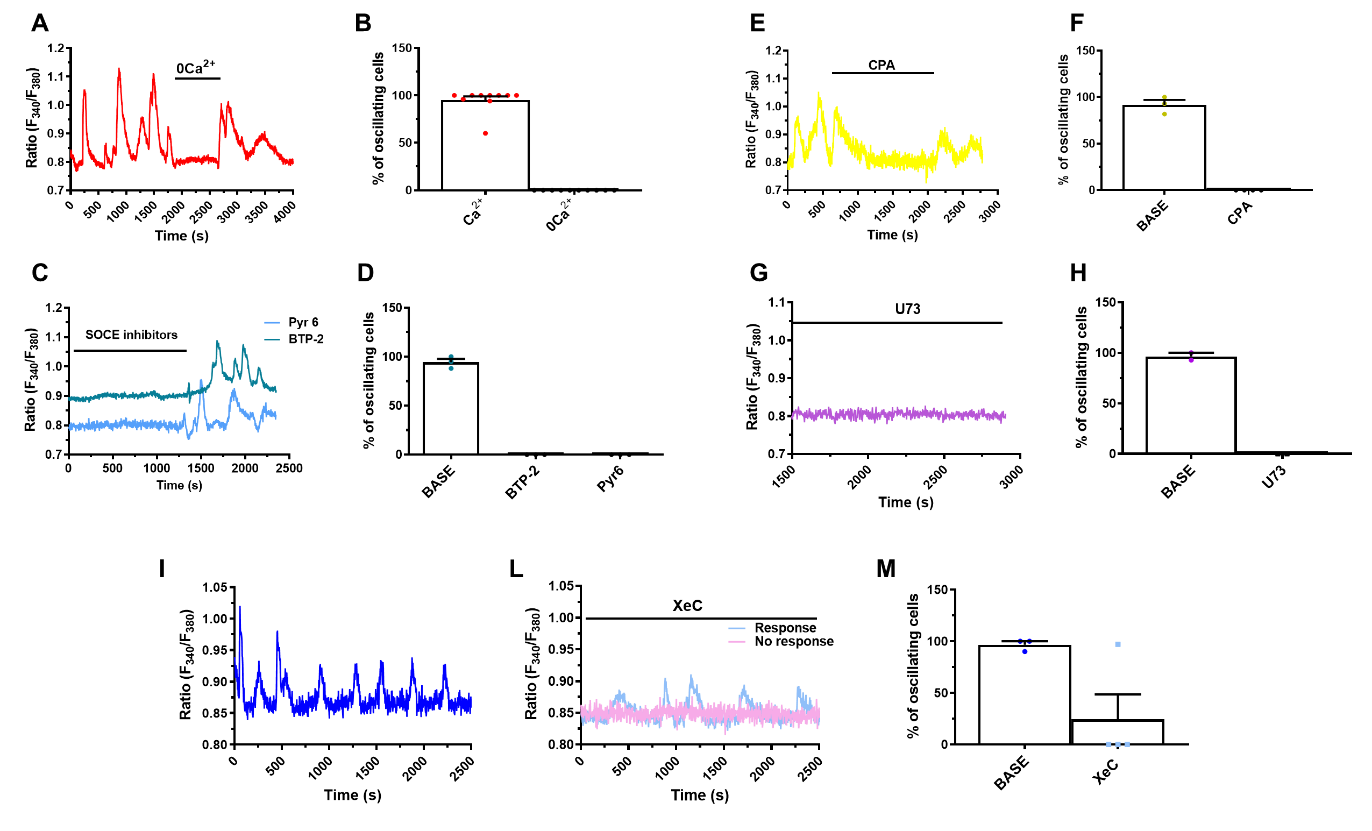


**Figure S4: SOCE and IP3Rs drive the spontaneous Ca^2+^ oscillations in ACM C-MSC.**

(**A**) Representative Ca^2+^ traces in C-MSC from ACM patients loaded with Fura-2/AM. The measurements were performed on unstimulated cells cultured in GM in presence of Ca^2+^ or in Ca^2+^-free solution (0Ca^2+^). **(B)** Quantification of peaks [Ca^2+^]_i_. The graphs represent the percentage of oscillating cells (n=491 cells).

**(C)** Representative Ca^2+^ traces in C-MSC from ACM patients loaded with Fura-2/AM. The measurements were performed on unstimulated cells cultured in GM in absence (BASE) or presence of two distinct ORAI1 inhibitors PYR6 (10 μM, 10 min) or BTP-2 (20 μM, 10 min;). **(D)** Quantification of peaks [Ca^2+^]_i_. The graphs represent the percentage of oscillating cells (n=100 cells BASE *vs.* n=155 cells BTP-2 *vs.* n=162 cells PYR6)

**(E)** Representative Ca^2+^ traces in C-MSC from ACM patients loaded with Fura-2/AM. The measurements were performed on unstimulated cells cultured in GM in absence (BASE) or presence of CPA (30 µM). **(F)** Quantification of peaks [Ca^2+^]_i_. The graphs represent the percentage of oscillating cells. (n=146 cells BASE *vs.* n=165 cells CPA).

**(G)** Representative Ca^2+^ traces in C-MSC from ACM patients loaded with Fura-2/AM. The measurements were performed on unstimulated cells cultured in GM in absence (BASE) or presence of U73122 (U73; 10 μM, 30 min;). **(H)** Quantification of [Ca^2+^]_i_ peaks. The graphs represent the percentage of oscillating cells. (n=92 cells BASE *vs.* n= 78 cells U73).

**(I-L)** Representative Ca^2+^ traces in C-MSC from ACM patients loaded with Fura-2/AM. The measurements were performed on unstimulated cells cultured in GM in absence (BASE; **I**) or presence of XestosponginC (XeC, 1 μM; **L**). **(M)** Quantification of [Ca^2+^]_i_ peaks. The graphs represent the percentage of oscillating cells. (n=76 BASE *vs.* n= 34 out of 144 cells XeC; Two-tailed Student’s t-tests).

Data information: mean+SEM.


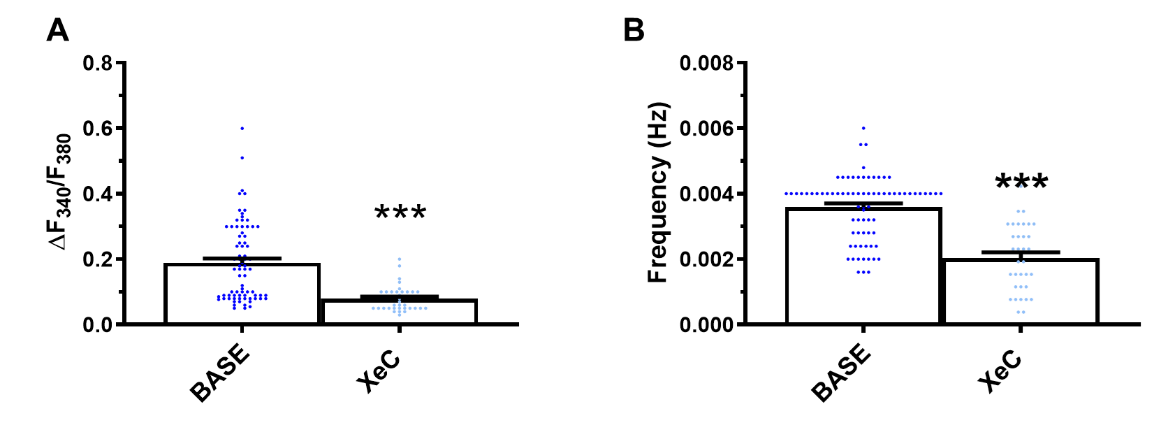


Fig. S5. Effect of XeC on spontaneous Ca^2+^ oscillations in ACM C-MSC.

**(A-B)** Quantification of [Ca^2+^]_i_ peaks in C-MSC from ACM patients loaded with Fura-2/AM. The measurements were performed on unstimulated cells cultured in GM in absence or presence of XestosponginC (XeC, 1 µM, 30 min). The graphs represent respectively: **(A)** oscillation frequency (BASE 0.1889+0.01374 a.u., n=76 out of 76 cells, *vs.* XeC 0.07882+0.006883 a.u., n=34 out of 144 cells; P<0.0001; Two-tailed Student’s t-tests); **(B)** oscillation frequency (BASE 0.003589+0.0001115 Hz, n=76 out of 76 cells, *vs.* XeC 0.002036+0.0001753 Hz, n=34 out of 144 cells; P<0.0001; Two-tailed Student’s t-tests).

Data information: mean+SEM. ***P < 0.001.


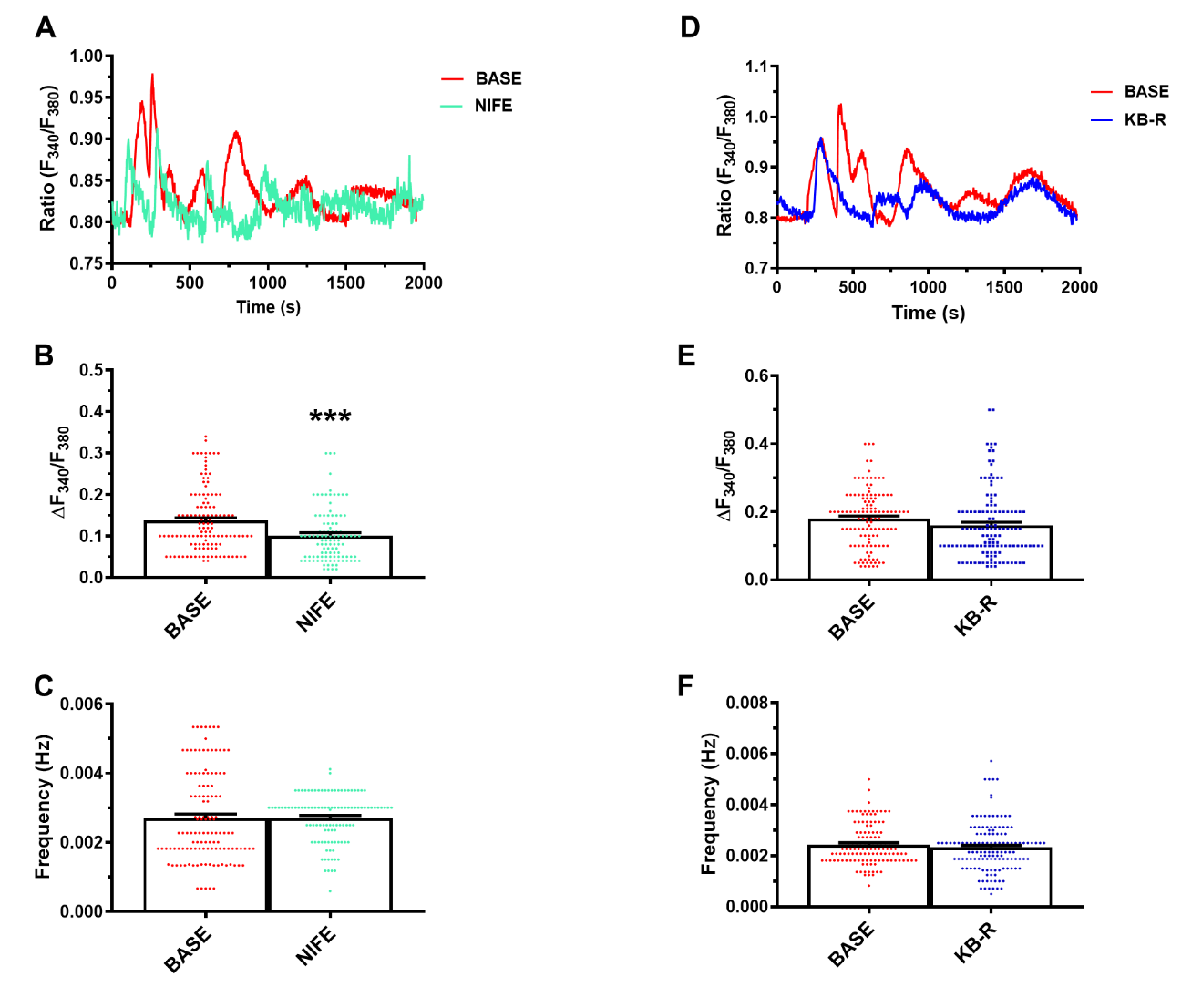


Fig. S6. Contribution of VOCC and reverse-mode NCX to spontaneous Ca^2+^ oscillations in ACM C-MSC.

**(A)** Representative Ca^2+^ traces in C-MSC from ACM patients loaded with Fura-2/AM. The measurements were performed on unstimulated cells cultured in GM in absence or presence of Nifedipine (NIFE, 10 µM, 30 min). **(B-C)** Quantification of [Ca^2+^]_i_ peaks. The graphs represent respectively: **(B)** oscillation amplitude **(**BASE 0.1373+0.006869 a.u., n=125, *vs*. NIFE 0.1013+0.006680 a.u., n=108; P=0.0002; Two-tailed Student’s t-tests**)**; **(C)** oscillation frequency **(**BASE 0.002704+0.0001154 Hz, n=125; *vs.* NIFE 0.002709+6.786e-005 Hz, n=108; not significant**).**

**(D)** Representative Ca^2+^ traces in C-MSC from ACM patients loaded with Fura-2/AM. The measurements were performed on unstimulated cells cultured in GM in absence or presence of KB-R (20 µM, 20 min). **(E-F)** Quantification of peaks [Ca^2+^]_i_. The graphs represent respectively: **(E)** oscillation amplitude **(**BASE 0.1801+0.007696 a.u., n=124, *vs.* KB-R 0.1606+0.008646 a.u., n=134; not significant**)**; **(F)** oscillation frequency **(**BASE 0.002440+7.129e-005 a.u., n=124, *vs.* KB-R 0.002327+8.378e-005, n=134; not significant**).**

Data information: mean+SEM. ****P* < 0.001.


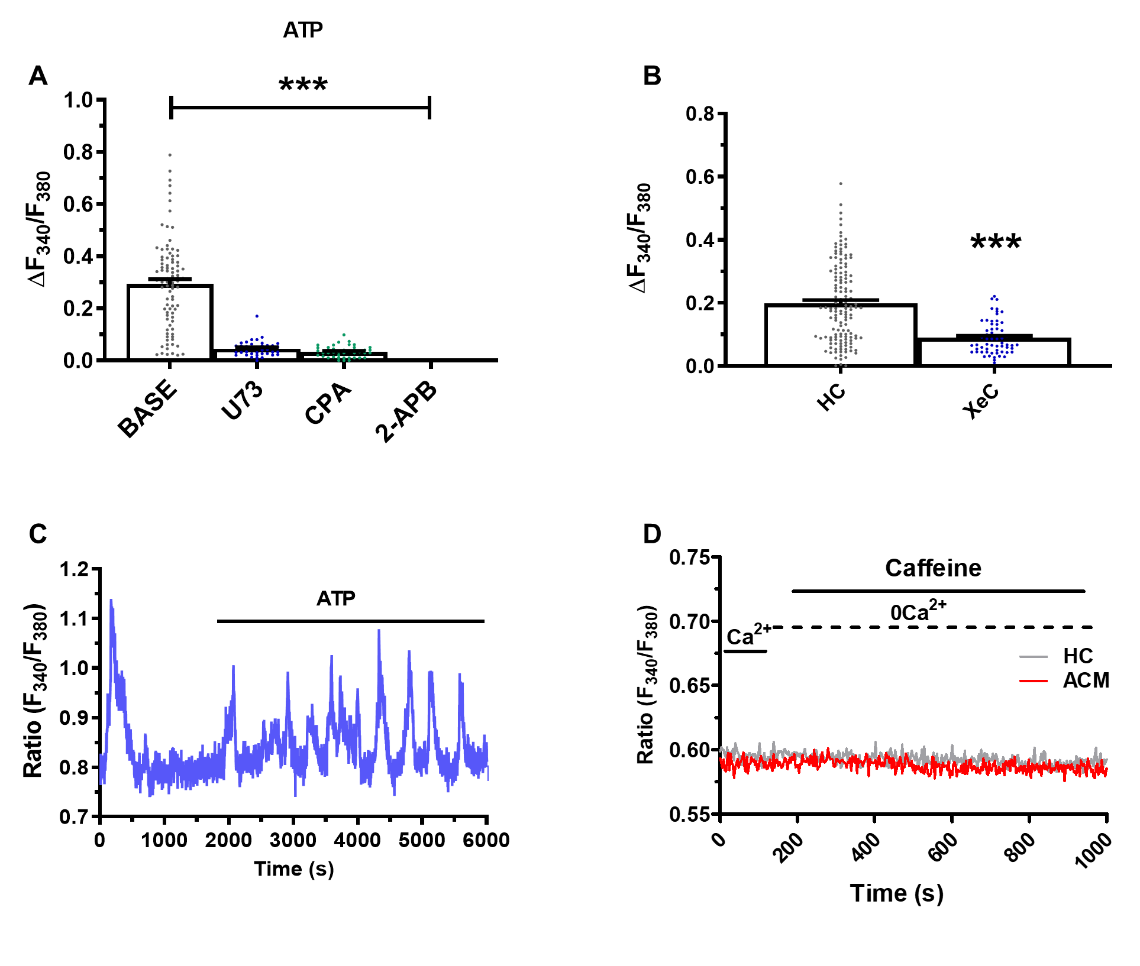


Fig. S7. IP3R, but not RYR, contribute to spontaneous Ca^2+^ oscillations in ACM C-MSC.

(A) Quantification of [Ca^2+^]_i_ peaks in C-MSC from ACM patients loaded with Fura-2/AM. The measurements were performed on unstimulated cells cultured in GM in absence (BASE) or presence of U73 (10 µM, 30 min), CPA (30 µM, 30 min) or 2-APB (50 µM, 30 min). The graphs represent the intracellular Ca^2+^ peak evoked by ATP (BASE 0.2940+0.01823 a.u., n=90 out of 90 cells, *vs.* U73 0.04571+0.004903 a.u., n=38 out of 83 cells; P< 0.0001; vs CPA 0.03383+0.003405, n=42 out of 90 cells; P< 0.0001; 2-APB no response, n=48; One-Way Anova test).

**(B)** Quantification of [Ca^2+^]_i_ peaks evoked by ATP. The graphs represent the oscillation amplitude (HC 0.1982+0.01062 a.u., n=141 out of 157 cells, vs. XeC 0.08917+0.006830a.u., n=60 out of 121 cells; P<0.0001; Two-tailed Student’s t-tests).

(C) Representative Ca^2+^ traces in C-MSC from ACM patients loaded with Fura-2/AM. The measurements were performed on cells cultured in GM and stimulated with low doses of ATP (10 µM).

(D) Representative Ca^2+^ traces in C-MSC from HC donors and ACM patients loaded with Fura-2/AM. The measurements were performed on cells cultured in GM and stimulated with caffeine (5 mM).

Data information: mean+SEM. ****P* < 0.0001.


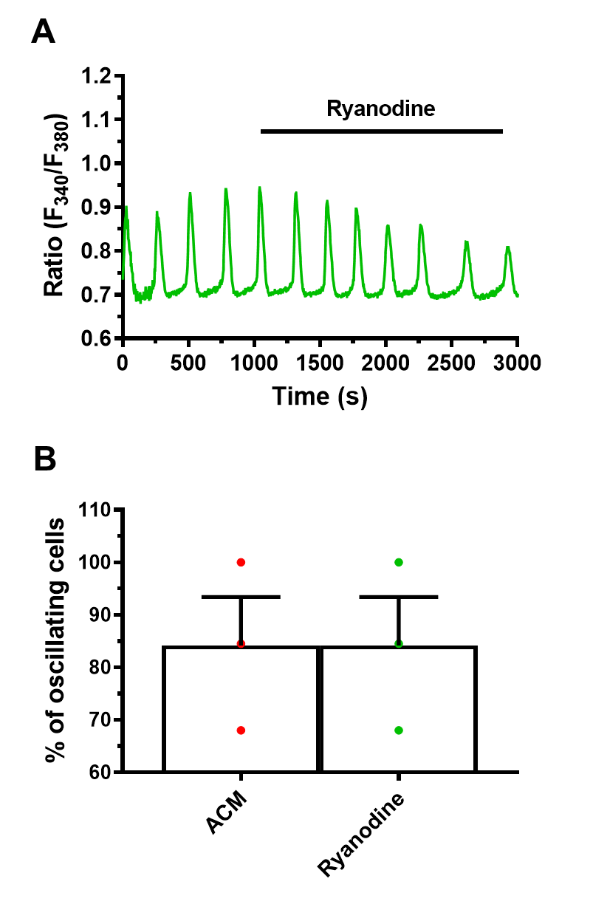


**Fig. S8. Ryanodine do not affect spontaneous Ca^2+^ oscillations in ACM C-MSC**

**(A)** Representative Ca^2+^ traces in C-MSC from ACM patients loaded with Fura-2/AM. The measurements were performed on cells cultured in GM in absence or presence of Ryanodine (10 µM).

**(B)** Quantification of [Ca^2+^]_i_ peaks. The graphs represent the percentage of oscillating cells. (ACM 84.17+9.239 a.u. vs. Ryanodine 84.17+9.239 a.u.; n=122 out of 145 cells from three different experimental series; not significant; Two-tailed Student’s t-tests).

Data information: mean+SEM.

**
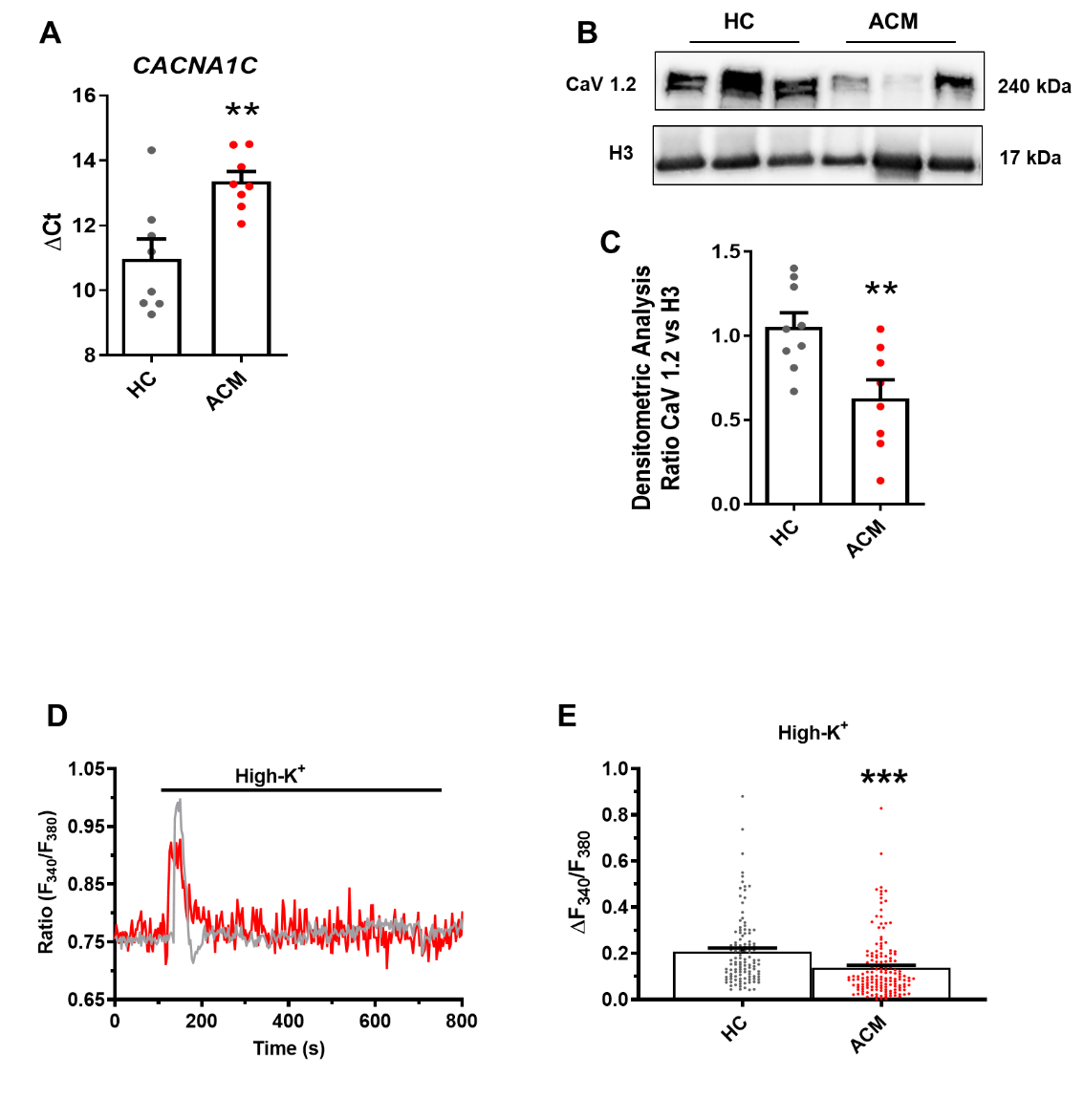
**

**Fig. S9. Voltage-Gated Ca^2+^ Entry is lower in ACM C-MSC.**

**(A)** Expression of *CACNA1C* in total RNA extracts of C-MSC from HC donors and ACM patients. GAPDH was used as a house-keeping gene and qRT-PCR data are presented as the genes threshold cycles (Ct) with respect to the housekeeping gene GAPDH (ΔCt) (*n=*8; HC 10.97+0.6111 *vs.* ACM 13.35+0.3069; *P=*0.0037; Two-tailed Student’s t-tests).

**(B)** Representative images of Western blot analysis of proteins extracted from HC and ACM C-MSC cultured in GM, hybridized with anti-CAV1.2 antibody. Immunostaining of the housekeeping H3 is shown for normalization. **(C)** Densitometric analysis of CAV1.2 (*n=*8; HC 1.052+0.08366 *vs.* ACM 0.6288+0.1094; *P=*0.0071; Two-tailed Student’s t-tests) levels, normalized on H3.

**(D)** Representative Ca^2+^ tracings showing the effect of High-K^+^ extracellular solution (100 mM KCl) on unstimulated C-MSC from HC donors and ACM patients loaded with Fura-2/AM. **(E)** Rate of amplitude of the intracellular Ca^2+^ response to High-K^+^ in C-MSC from HC donors and ACM patients (HC 0.2087+0.01402 a.u., n=115, vs. ACM 0.1379+0.01064, n=155; P<0.0001; Two-tailed Student’s t-tests).

Data information: mean+SEM. **P* < 0.05, ***P* < 0.01 and ****P* < 0.0001


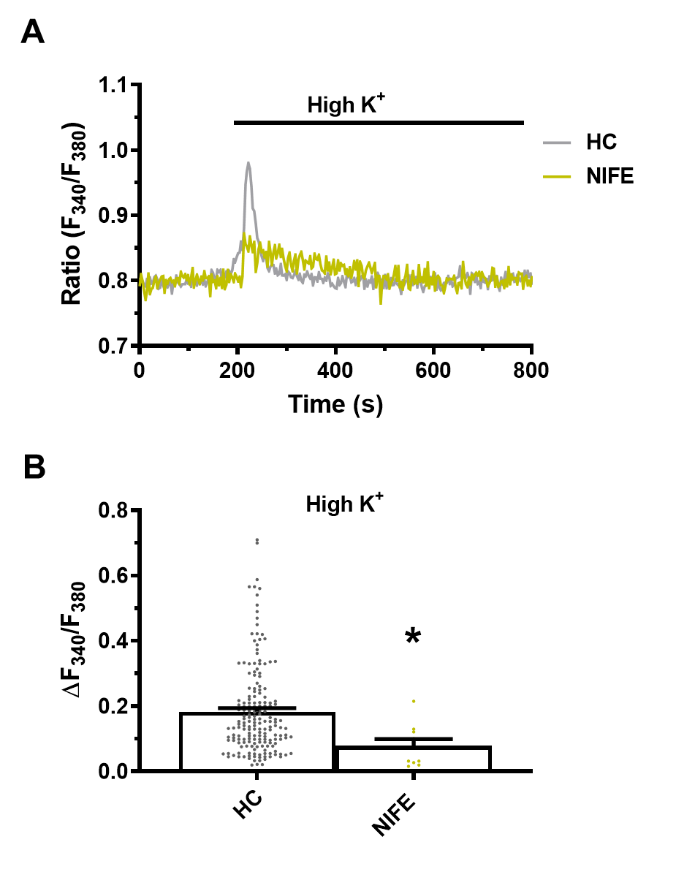


**Fig. S10. Nifedipine inhibits Ca^2+^ response to High K^+^**

**(A)** Representative Ca^2+^ traces in C-MSC from HC donors loaded with Fura-2/AM. The measurements were performed on cells cultured in GM and stimulated High-K^+^ extracellular solution (100 mM KCl) in absence or presence of Nifedipine (NIFE 10 µM).

**(B)** Quantification of [Ca^2+^]_i_ peaks evoked by High-K^+^. The graphs represent the oscillation amplitude (HC 0.1833+0.01022 a.u., n=180 out of 208 cells, vs. NIFE 0.07830+0.02055 a.u., n=10 out of 53 cells; P= 0,0173; Two-tailed Student’s t-tests).

Data information: mean+SEM. **P* < 0.05

**
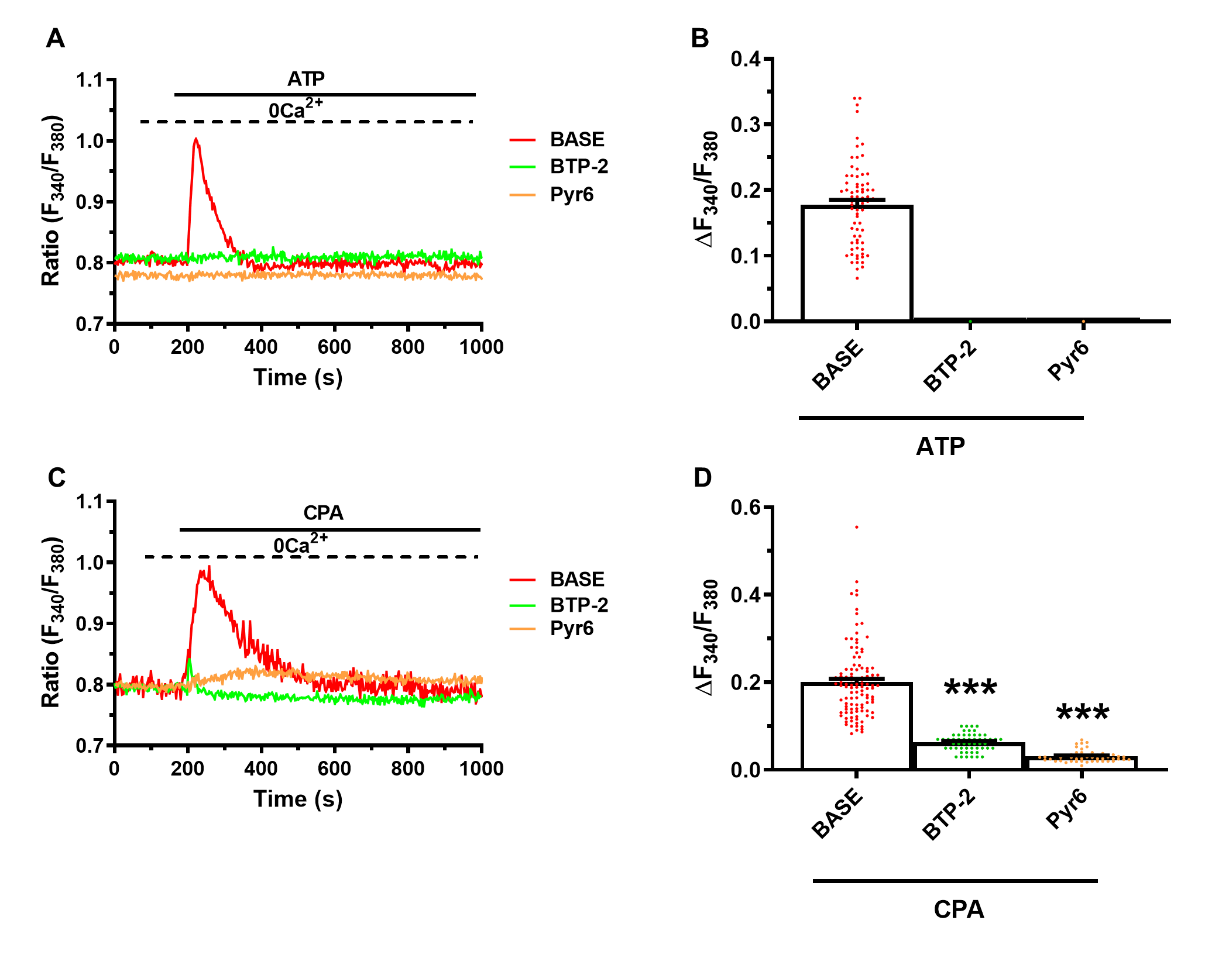
**

**Fig. S11.** **Blocking constitutive SOCE prevents ER Ca^2+^ release in ACM C-MSC.**

**(A)** Representative Ca^2+^ traces obtained from C-MSC deriving from ACM patients loaded with Fura-2/AM and challenged with ATP. Intracellular Ca^2+^ release was evoked by ATP (100 µM) in the absence (BASE) and presence of two distinct ORAI1 inhibitors, i.e., BTP-2 (20 μM, 20 min) or PYR6 (10 μM, 10 min). **(B)** Rate of amplitude of ATP-evoked Ca^2+^ mobilization (BASE 0.1780+0.007334 a.u., n=75 out of 75 cells, *vs.* BTP-2 no response, n=59; vs PYR6 no response, n=67). **(C)** Representative Ca^2+^ traces obtained from C-MSC deriving from ACM patients loaded with Fura-2/AM and challenged with CPA. Intracellular Ca^2+^ release was evoked by CPA (30 µM) in the absence (BASE) and presence of two distinct ORAI1 inhibitors, i.e., BTP-2 (20 μM, 20 min) or PYR6 (10 μM, 10 min). **(D)** Rate of the amplitude of CPA-evoked Ca^2+^ mobilization (BASE 0.2005+0.007805 a.u., n=111 out of 111 cells, *vs.* BTP-2 0.06296+0.002691 a.u., n=54 out of 114 cells; P< 0.0001; *vs.* PYR6 0.03113+0.001907, n=48 out of 48 cells; P< 0.0001; One-Way Anova).

Data information: mean+SEM. ****P* < 0.0001.


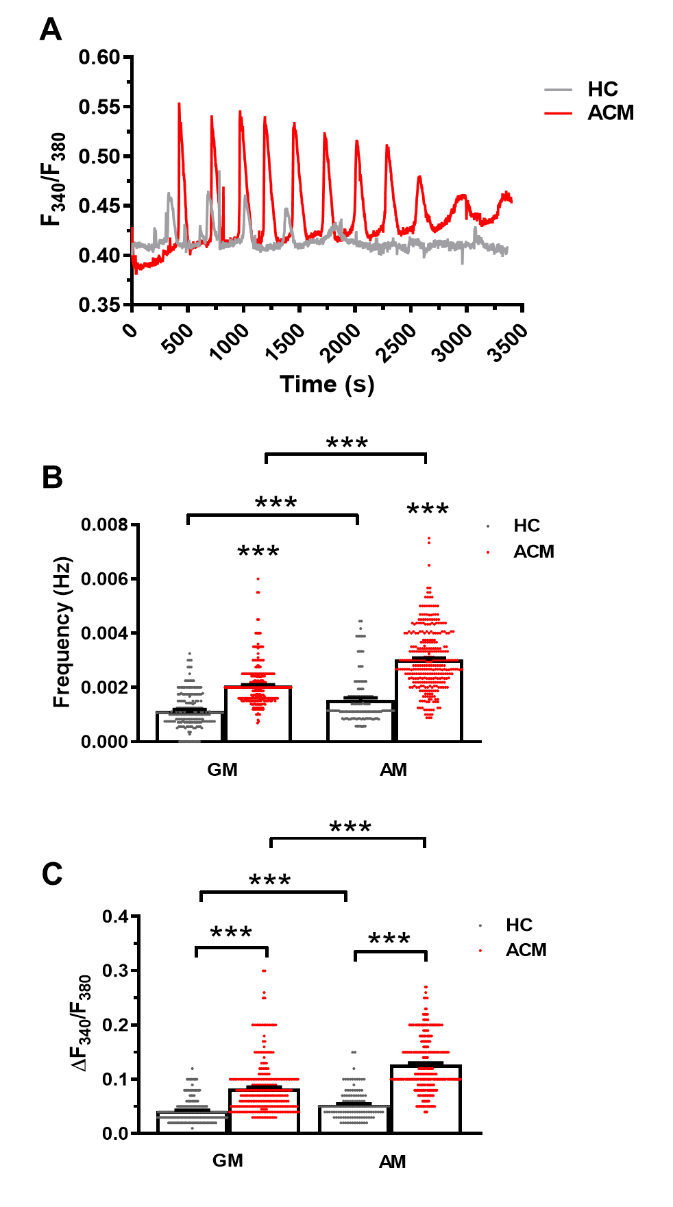


Fig. S12. Spontaneous Ca^2+^ oscillations during adipogenesis.

**(A)** Representative Ca^2+^ traces in C-MSC from HC donors and ACM patients loaded with Fura-2/AM. **(B-C)** Quantification of [Ca^2+^]_i_ peaks in C-MSC from HC donors and ACM patients. The measurements were performed on unstimulated cells cultured in GM or AM for 3 days. The graphs represent respectively: **(B)** oscillations frequency (HC GM 0.001146+4.388e-005 Hz, n=208, *vs.* ACM GM 0.002062+4.175e-005 Hz, n=307; P<0.0001; HC AM 0.001535+7.905e-005 Hz, n=130 *vs.* ACM AM 0.003031+6.194e-005, n=327; P<0.0001; HC GM 0.001146+ 4.388e-005, n=208 *vs.* HC AM 0.001535+7.905e-005, n=130; P<0.0001; ACM GM 0.002062+4.175e-005, n=307 *vs.* ACM AM 0.003031+6.194e-005, n=327; P<0.0001; Two-Way Anova test); **(C)** oscillation amplitude (HC GM 0.04191+0.001512 a.u., n=194, *vs.* ACM GM 0.08303+0.002734 a.u., n=307; P<0.0001; HC AM 0.05300+0.002232 a.u., n=130, *vs.* ACM AM 0.1276+0.002795 a.u., n=327; P<0.0001; HC GM 0.04191+0.001512, n=208 *vs.* HC AM 0.05300+0.002232, n=130; P<0.0001; ACM GM 0.08303+0.002734, n=307 *vs.* ACM AM 0.1276+0.002795, n=327; P<0.0001; Two-Way Anova test).

Data information: mean+SEM. ****P* < 0.0001.


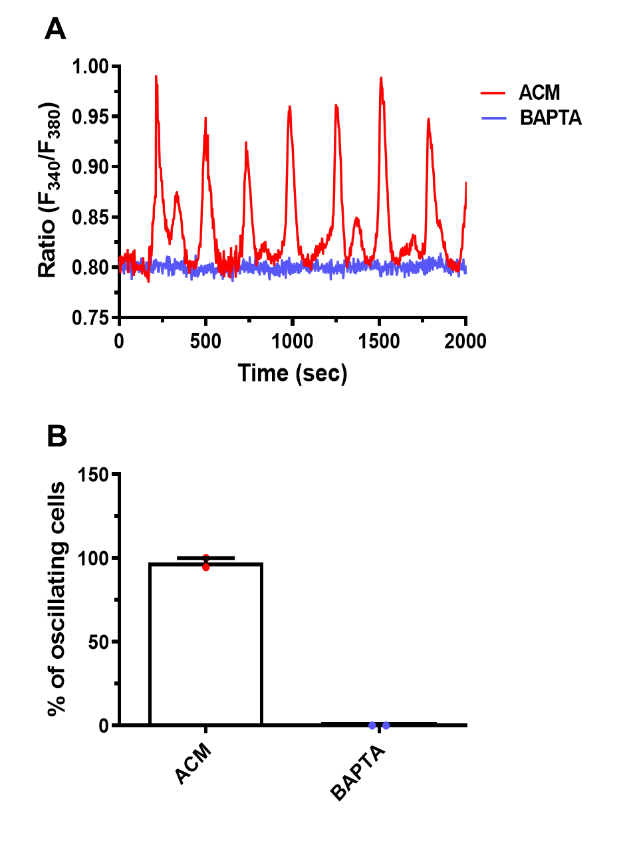


**Fig. S13. BAPTA inhibit the spontaneous Ca^2+^ activity of ACM C-MSC.**

**(A)** Representative Ca^2+^ traces in C-MSC from ACM patients loaded with Fura-2/AM. The measurements were performed on cells cultured in GM in absence or presence of BAPTA (10 µM). **(B)** Quantification of peaks [Ca^2+^]_i_. The graphs represent the percentage of oscillating cells. (ACM 97.30+2.700 a.u. vs. BAPTA no response a.u.; n=82 out of 85 cells from two different experimental series; not significant; Two-tailed Student’s t-tests).

Data information: mean+SEM.


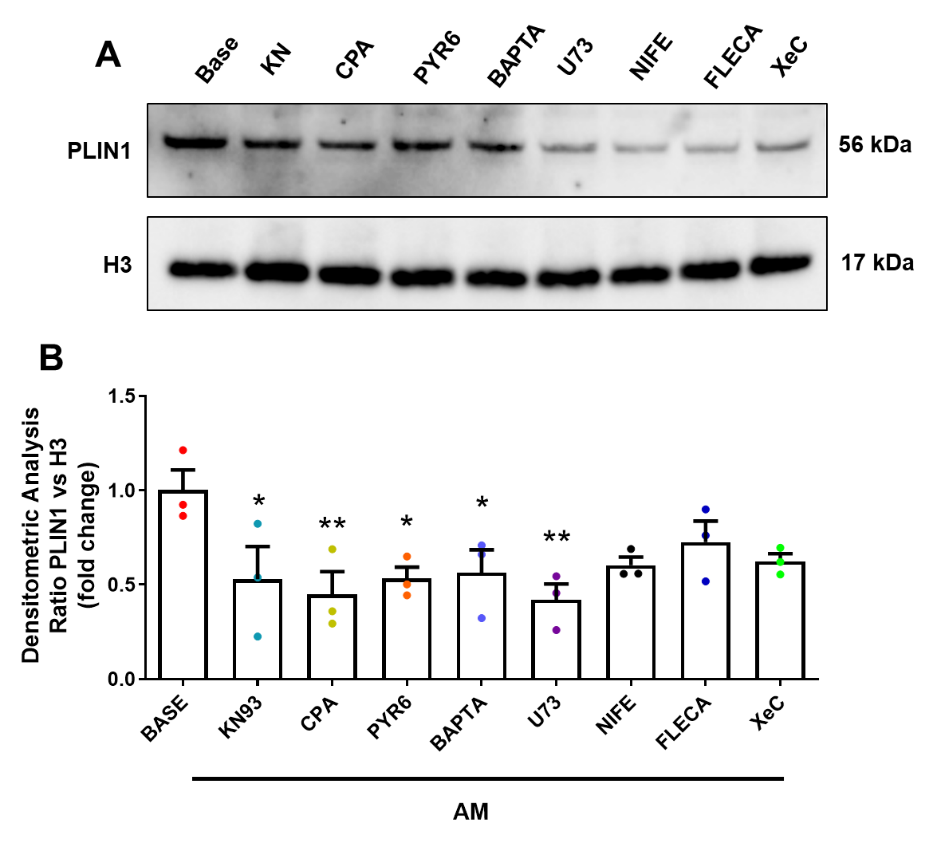


Fig. S14. PLIN1 modulation in ACM C-MSC.

**(A)** Representative images of Western blot analysis of proteins extracted from ACM C-MSC cultured in AM for 3 days supplemented or not with [Ca^2+^]_i_ modulators or CaMKII inhibitor (KN93 10 µM, CPA 30 µM, PYR6 10 µM, BAPTA 30 µM, U73 10 µM, NIFE 10 µM, FLECA 10 μM and XeC 1 μM; 3 days), hybridized with anti-PLIN1 antibody. Immunostaining of the housekeeping H3 is shown for normalization. **(B)** Densitometric analysis of PLIN1 levels, normalized on H3 (n=3; BASE 1.000+0.1076 *vs.* KN93 0.5288+0.1726 (*P=*0.0294); *vs.* CPA 0.4470+0.1221 (*P=*0.0091); *vs.* PYR6 0.5314+0.06132 (*P=*0.0305); *vs.* BAPTA 0.5637+0.1211 (*P=*0.0476); *vs.* U73 0.4199+0.08401 (*P=*0.0062); *vs.* NIFE 0.6019+0.04375 (*P=*0.0794); *vs.* FLECA 0.7255+0.1114 (*P=*0.3426); *vs.* XeC 0.6234+0.04104 (*P=*0.1048); one-way ANOVA test). The value is shown for each condition relative to the values BASE.

Data information: mean+SEM. **P* < 0.05 and ***P* < 0.01.


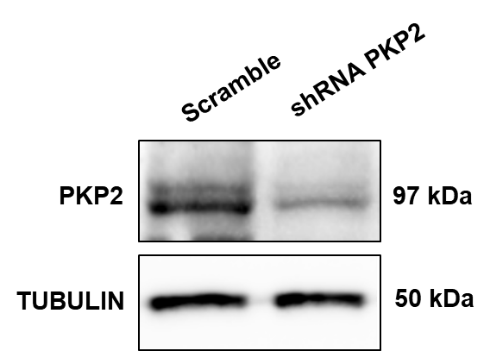


Fig. S15. PKP2 silencing in C-MSC.

Representative images of Western blot analysis of proteins extracted from HC C-MSC treated with scramble siRNA or PKP2 cultured in GM, hybridized with PKP2 antibody. Immunostaining of the housekeeping Tubulin are shown for normalization.

|  | **Sex/Age**  **(at recruitment)** | **Age/Type of first manifestation** | **Dysfunction/ structural alterations at imaging** | **Tissue characterization** | **Repolarization abnormalities** | **Depolarization conduction**  **abnormalities** | **Arrhythmias** | **Family history/ Genetics** |
| --- | --- | --- | --- | --- | --- | --- | --- | --- |
| ACM1 | M/ 52 | 51/ VT | minor | minor | minor | minor | major | major  (*PKP2* c.2013delC p.Lys672ArgfsX12) |
| ACM2 | M/ 42 | 42/VT | major | not conclusive | major | negative | major | major  (*PKP2* c.1643delG p.Gly548ValfsX15) |
| ACM3 | M/ 41 | 27/PVCs | major | not conclusive | major | negative | minor | major  (*PKP2* c.2013delC p.Lys672ArgfsX12) |
| ACM4 | M/ 46 | 45/syncope | minor | not conclusive | minor | negative | negative | Major (*DSG2* c.1003A>G p.Thr335Ala) |
| ACM5 | M/ 43 | 43/VT | major | minor | minor | minor | major | negative |
| ACM6 | F/ 52 | 50/PVCs | major | major | minor | minor | minor | negative |
| ACM7 | M/ 41 | 34/PVCs | major | minor | negative | minor | minor | Not conclusive  *PKP2* VUS c.548G>A p.Ser183Asn |
| ACM8 | F/ 41 | 38/syncope | minor | major | minor | negative | minor | negative / not available |
| ACM9 | M/ 49 | 46/VT | major | not conclusive | major | negative | major | major  *DSC2* homozygous  c.2398-2399insG  p.A800GfsX37 |
| ACM10 | F/ 39 | 35/ VT | minor | not conclusive | negative | minor | major | major  *PKP2*: c.2013delC, p.Lys672ArgfsX12 |
| ACM11 | F/ 19 | 19/VF | major | major | major | negative | major | negative / not available |
| ACM12 | M/ 38 | 26/VT | minor | not conclusive | major | negative | major | negative / not available |
| ACM13 | M/ 54 | 54/syncope | minor | not conclusive | minor | negative | major | negative / not available |
| ACM14 | M/ 69 | 56/ PVCs | minor | not conclusive | major | negative | minor | negative / not available |
| ACM15 | F/ 28 | 23/VT | minor | minor | minor | minor | minor | negative / not available |

Table S1. Clinical data of ACM patients enrolled for biopsy samples.

Minor and major scores are given according to the International Task Force Criteria for the diagnosis of ACM (5). VT: ventricular tachycardia; PVCs: premature ventricular contractions; VF: ventricular fibrillaton. Mutations are reported only when considered pathogenic or likely pathogenic.

| **ID** | **Sex (M=male; F=female)** | **Age** | **Cause of death** | **Concomitant diseases** | **Drugs** | **Cardiovascular risk factors** |
| --- | --- | --- | --- | --- | --- | --- |
| HC1 | M | 51 | Cerebral hemorrhage | / | / | Hypertension |
| HC2 | M | 42 | Multiple trauma | / | / | / |
| HC3 | M | 41 | Multiple trauma | / | / | / |
| HC4 | M | 50 | Multiple trauma | / | / | / |
| HC5 | M | 44 | Cerebral hemorrhage | / | / | / |
| HC6 | F | 58 | Cerebral hemorrhage | / | / | Smoking, Hypertension |
| HC7 | M | 40 | Cerebral hemorrhage | / | / | Smoking, Hypertension |
| HC8 | F | 48 | Cerebral hemorrhage | / | / | Hypertension |
| HC9 | M | 49 | Cerebral hemorrhage | / | / | / |
| HC10 | F | 50 | Respiratory failure | Idiopathic pulmonary fibrosis | Angiotensin Receptor Blockers | / |
| HC11 | F | 18 | Multiple trauma | / | / | / |
| HC12 | M | 33 | Cerebral hemorrhage | / | / | / |
| HC13 | M | 55 | Cerebral hemorrhage | / | / | / |
| HC14 | M | 57 | Cerebral hemorrhage | / | / | / |
| HC15 | F | 46 | Cerebral hemorrhage | / | / | Smoking |

**Table S2. Clinical features of the deceased tissue donors (with healthy heart) enrolled in this study.**

| **Gene** | **Forward primer** | **Reverse primer** |
| --- | --- | --- |
| *CAMK2A* | GAA GAG CGA TGG TGT GAA GA | ACT TTG GTG TCT TCA TCC TCG |
| *CAMK2B* | CTC TCG CCA CAA TGT CTT CA | CTG TCA GCC AGA GAT CAC CA |
| *CAMK2C* | TCC GAG CCT CAC GTT CTA GT | CCG ACG ACT ACC AGC TCT TC |
| *CAMK2D* | GGC TGC TGA GAA ATT CCT TG | ACT ATC AAC CCT GCC AAA CG |
| *ATP2A2* | CGAACCCTTGCCACTCATCT | CCAGTATTGCAGGTTCCAGGT |
| *ITPR2* | ACCTTGGG GTTAGTGGATGA | CCTTGTTTGGCTTGCTTTGC |
| *ORAI1* | AGTTACTCCGAGGTGATGAG | ATGCAGGTGCTGATCATGAG |
| *STIM1* | CCTCAGTATGAGGAGACCTT | TCCTGAAGGTCATGCAGACT |
| *CACNA1C* | AACAACAGGTTTCGCCTCCA | GTTCCTGAAGGAGGTGTGCT |
| *RYR2* | CTAATGTCTGGGTGGGCTGG | TGCTGCGTTTGATGCTTTCA |
| *PLN* | ATCACAGCTGCCAAGGCTAC | AGCTGAGCGAGTGAGGTATTG |
| *SCN5A* | GCCCTCCTCAGCCCCATCCTC | GGCATCGGCAAAGTCAGACAG |
| *GAPDH* | ATGTTCGTCATGGGTGTGAA | GTCTTCTGGGTGGCAGTGAT |

Table S3. Primer sequences 5’ - 3’.

| **Protein** | **Clonality/Code** | **Source** | **Company** | **Diluition** |
| --- | --- | --- | --- | --- |
| phospho-CaMKII (T286) | Polyclonal, ab32678 | Rabbit | Abcam | WB: 1:1000 |
| CaMKII | Monoclonal, sc-5306 | Mouse | Santa Cruz | WB: 1:500 |
| SERCA2 ATPase | Monoclonal, ab2861 | Mouse | Abcam | WB: 1:1000 |
| IP3R | Monoclonal, sc-377518 | Mouse | Santa Cruz | WB: 1:1000 |
| STIM1 | Monoclonal, #5668 | Rabbit | Cell Signaling | WB: 1:1000 |
| ORAI1 | Polyclonal, sc-68895 | Rabbit | Santa Cruz | WB: 1:1000 |
| CACNA1C | Monoclonal, ab84814 | Mouse | Abcam | WB: 1:1000 |
| Ryanodine Receptor | Monoclonal, MA3-916 | Mouse | Invitrogen | WB: 1:1000 |
| Na^+^ CP type Vα | Monoclonal, sc-271255 | Mouse | Santa Cruz | WB: 1:100 |
| PLIN1 | Polyclonal, BP5015 | Guinea Pig | Origene | WB: 1:1000 |
| PKP2 | Monoclonal, 610788 | Mouse | BD Biosciences | WB: 1:1000 |
| COL1A1 | Monoclonal, #84336 | Rabbit | Cell Signaling | IF: 1:200 |
| H3 | Polyclonal, #9715 | Rabbit | Cell Signaling | WB: 1:1000 |
| GAPDH | Polyclonal, sc-25778 | Rabbit | Santa Cruz | WB: 1:1000 |
| TUBULIN | Monoclonal, ab179513 | Rabbit | Abcam | WB: 1:1000 |

Table S4. Primary antibodies.

**References**

1. Prakriya M, Lewis RS. Store-Operated Calcium Channels. Physiological reviews. 2015;95(4):1383-436.

2. Balducci V, Faris P, Balbi C, Costa A, Negri S, Rosti V, et al. The human amniotic fluid stem cell secretome triggers intracellular Ca(2+) oscillations, NF-kappaB nuclear translocation and tube formation in human endothelial colony-forming cells. J Cell Mol Med. 2021;25(16):8074-86.

3. Tan YZ, Fei DD, He XN, Dai JM, Xu RC, Xu XY, et al. L-type voltage-gated calcium channels in stem cells and tissue engineering. Cell proliferation. 2019;52(4):e12623.

4. Peng H, Hao Y, Mousawi F, Roger S, Li J, Sim JA, et al. Purinergic and Store-Operated Ca(2+) Signaling Mechanisms in Mesenchymal Stem Cells and Their Roles in ATP-Induced Stimulation of Cell Migration. Stem Cells. 2016;34(8):2102-14.

5. Marcus FI, McKenna WJ, Sherrill D, Basso C, Bauce B, Bluemke DA, et al. Diagnosis of arrhythmogenic right ventricular cardiomyopathy/dysplasia: proposed modification of the task force criteria. Circulation. 2010;121(13):1533-41.
